# Assessment of net knee moment-angle characteristics by instrumented hand-held dynamometry in children with spastic cerebral palsy and typically developing children

**DOI:** 10.1186/s12984-015-0056-y

**Published:** 2015-08-15

**Authors:** Helga Haberfehlner, Huub Maas, Jaap Harlaar, Irene E. Newsum, Jules G. Becher, Annemieke I. Buizer, Richard T. Jaspers

**Affiliations:** Faculty of Human Movement Sciences, VU University Amsterdam, Van der Boechorststraat 9, 1081 BT Amsterdam, The Netherlands; Department of Rehabilitation Medicine, VU University Medical Center, De Boelelaan 1118, 1081 HZ Amsterdam, The Netherlands; MOVE Research Institute Amsterdam, VU University Amsterdam, Amsterdam, The Netherlands, Van der Boechorststraat 9, 1081 BT Amsterdam, The Netherlands

**Keywords:** Cerebral palsy, Knee, Hamstrings, Muscle stiffness, Muscle contracture, Moment-angle characteristics, Popliteal angle, Muscle spasticity

## Abstract

**Background:**

The limited range of motion during walking in children with spastic cerebral palsy (SCP) may be the result of altered mechanical characteristics of muscles and connective tissues around the knee joint. Measurement of static net knee moment-angle relation will provide insights into these alterations, for which instrumented hand-held dynamometry may be applied.

The aims of this study were: (1) to test the measurement error of the estimated net knee moment-angle characteristics, (2) to determine the correlation between knee extension angle measurement at a standardized knee moment and popliteal angle from common physical examination and (3) to compare net knee moment–angle characteristics in SCP versus typically developing children.

**Methods:**

With the child lying in sideward position, the knee was extended by moving the lower leg by a hand-held force transducer on a low friction cart. Force data were collected for a range of knee angles. Data were excluded when activity (EMG) levels of knee extensor and flexor muscles exceeded the EMG level during rest by more than two standard deviations. The net knee flexion moments were calculated from recorded force data and measured moment arm. Reliability for knee angles corresponding with 0.5, 1, 2, 3, and 4 Nm knee net flexion moments was assessed by standard error of measurements (SEM) and smallest detectable difference (SDD).

**Results:**

For between day comparison, SEMs were about 5° and SDDs were below 14° for knee angles at 1-4 Nm net knee flexion moments. In SCP children, the knee angle measured at 4 Nm knee flexion moment was not related to the popliteal angle (r = 0.52). The slope at 4 Nm of the knee moment-angle curve in SCP children was significantly higher than that in typically developing children.

**Conclusions:**

The presented knee hand-held dynamometry allows assessment of net knee flexion moment-knee angle characteristics in typically developing and SCP children and can be used to identify clinically relevant changes as a result of treatment. Overall stiffness of structures that contribute to the net knee flexion moment at the knee (i.e. muscles, tendons, ligaments) is elevated in SCP children.

**Electronic supplementary material:**

The online version of this article (doi:10.1186/s12984-015-0056-y) contains supplementary material, which is available to authorized users.

## Background

Cerebral palsy is a neurological disorder which involves impaired muscle function that restricts the ability to move and to maintain posture, causing limitations in daily activities [1]. Cerebral palsy is the most common physically disabling condition in childhood [2]. In children with cerebral palsy, the prevalence of the spastic motor disorder is high [3]. The level of daily limitations varies substantially, ranging from children who only show limitations in sports activities to children who have no independent mobility [4]. In children with spastic cerebral palsy (SCP), crouch gait is a common gait deviation, which is characterized by excessive hip and knee flexion during stance [5–7]. This gait pattern is often associated with a lack of knee extension in terminal swing, which restrains step length [8]. Weakness of the soleus muscle has been described as a major risk factor for the development of crouch gait [7]. In addition, children may develop hamstring muscle contractures (i.e. a high resistance to knee extension when the hip is kept in a flexed position) with or without a knee-joint extension limitation [7]. The mechanisms underlying the etiology of hamstrings contracture and knee extension limitations and how mechanical tissue properties besides alterations in muscle excitation contribute to crouch gait in SCP children is largely unknown.

Part of the children with SCP walking in crouch gait need surgical lengthening of the hamstring muscle-tendon complex (MTC) [9]. In most cases, such an intervention is effective in improving gait [10, 11]. However, recurrence and reoperation rates are substantial [10]. Also, there are reports of overcorrection of the hamstring muscles leading to a hyperextension of the knee and an increase in lumbar lordosis and anterior pelvic tilt [10, 11].

In clinical practice, resistance to knee extension due to muscle, ligament and joint stiffness is estimated by the popliteal angle [12] and is used as indication for surgical lengthening. The popliteal angle is measured with the ipsilateral hip hold flexed at 90° while the knee is passively extended to the knee angle at which increased resistance is perceived. The contralateral hip is kept fully extended at 0° [12]. Assessment of the popliteal angle, despite being widely used, has some methodological limitations: (1) The maximal attainable knee joint angle is subjectively determined, (2) correct position of hip and pelvis is difficult to maintain manually during popliteal angle measurements and (3) the variability of popliteal angle measurements is affected by voluntary and/or involuntary muscle activation [13–16].

In previous research it has been emphasized that the current indication of surgical lengthening of hamstring muscle based on popliteal angle may be insufficient and that there is a need of estimation of muscle length to indicate surgical lengthening [17–19]. Muscle length is currently estimated using musculoskeletal modelling, without individual morphological data [17–19].

An approach that allows to measure morphological determinants of the length-force characteristics directly in relation to altered mechanical behaviour of the joint, may increase our knowledge of mechanisms underlying limitations in range of motion (ROM) in SCP. Morphological determinants of the length-force characteristics involve muscle belly length, fascicle length, physiological cross-sectional area and pennation angle; all of which can be measured by ultrasound imaging [20]. For the ankle joint such an approach has been developed [21–23]. However, such an approach has not previously been described for the knee joint.

A first step towards a similar approach for the knee is to determine the mechanical properties of tissues around the knee by measuring net knee moment as a function of the knee angle over the full ROM [24]. Net knee flexion moment-angle curves were measured in healthy adults with instrumented hand-held dynamometry at standardized low muscle activation [25, 26], but to our knowledge neither in SCP nor TD children. In addition, available methods for assessment of net knee moment-angle characteristics have not been evaluated for their precision i.e. measurement error [25, 26].

Therefore, the aims of this study were: (1) to present a hand-held dynamometry approach and to determine its measurement error for assessment of net knee moment-angle characteristics in SCP and TD children in repeated measurements, (2) to determine the correlation with popliteal angle and (3) to compare net knee moment-angle characteristics between SCP and TD children.

## Methods

### Study population

The study protocol was approved by the Medical Ethics Committee of the VU University Medical Center (VUmc), Amsterdam (The Netherlands). All children and their parents gave their written informed consent.

Children with SCP were recruited from a group of children with spastic cerebral palsy (SCP) who were under medical treatment in the VUmc from April to October 2012. Patients included had: (1) a clinical diagnosis of SCP [1, 2], (2) a Gross Motor Function Classification System (GMFCS) Expanded and Revised Class I-III [27] and (3) were 8-16 years old. Patients that were currently involved in treatment that could affect the structural properties of the hamstring muscles were excluded. Specifically, this includes (1) medication that influences neuromuscular properties, treatment with Botulinum toxin A or 24-h casting within three months before measurements or (2) selective dorsal rhizotomy or surgery within one year prior to measurements. The control group consisted of age matched TD children.

### Experimental protocol

**Fig. 1:**
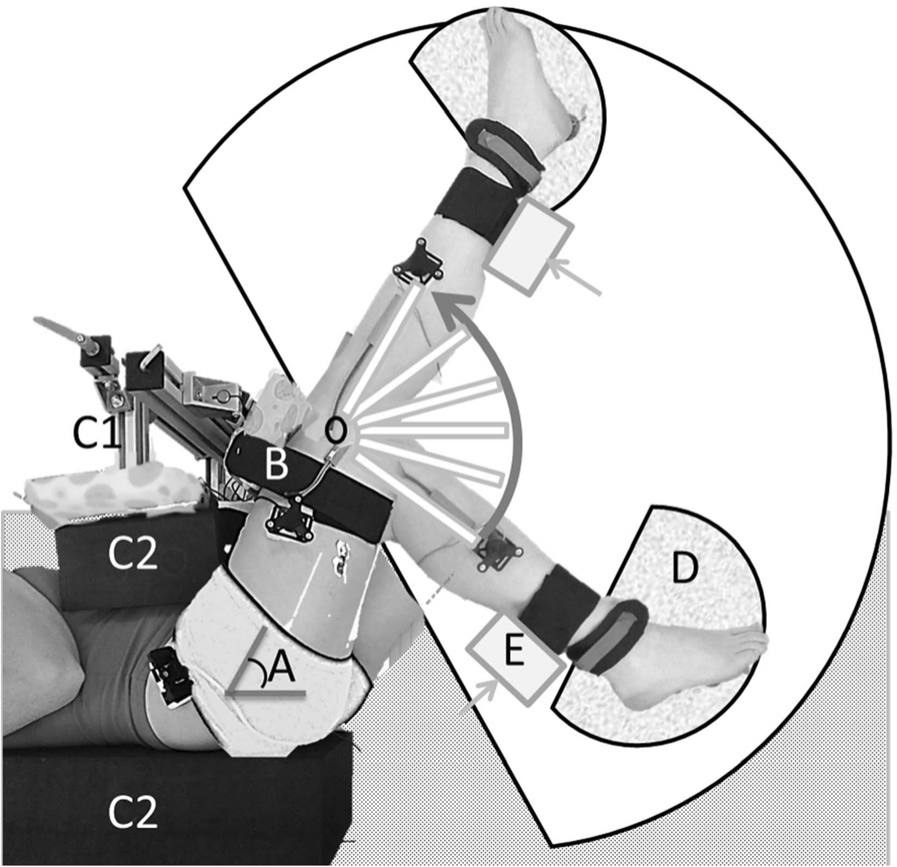
Top view of hand-held dynamometry measurement setup. Children were positioned on their left side on a treatment table, with the hip of the measured (right) leg at 70° flexion (*a*). Pelvis and upper leg were tightly secured – the upper leg with a bandage (*b*) and the pelvis with an adjustable frame (*c1*) and foam blocks on both sides of the trunk (*c2*). The lower leg was positioned on a low-friction movable plate (*d*). The lower leg was manually moved with a hand-held force transducer (*e*) through its range of motion with stops for 10 s every 5°

Subjects were lying on their left side on a comfortable examination table. The right leg was measured with the right hip positioned in 70° flexion. The left hip was put in a comfortable slightly flexed position (20°-40°). To prevent pelvic tilt and hip movement during measurements, pelvis and upper leg were tightly secured to the setup - the pelvis with an adjustable frame and foam blocks on both sides of the trunk and the upper leg with a bandage. The right lower leg was resting on a low friction cart (appropriately fastened) with the ankle in plantar flexion (about -20°) to minimize effects of gastrocnemius on knee moment (Fig. 1). This setup was designed such that in future experiments simultaneous ultrasound imaging of medial hamstrings would be possible.

Changes in knee angle were measured using a Twin Axis Goniometer SG150 (Biometrics Ltd, UK) of which one part was placed on the lateral side of the upper leg along the line between greater trochanter and the lateral condyle of the femur. The other part was placed on the lower leg along the line between the caput fibulae and the lateral malleolus [28]. The goniometer was attached to the skin when the knee was in 90° flexion. Technical clusters of three markers (Optotrak, NDI, Canada) were attached to verify whether movement of upper leg or pelvis occurred. Use of anatomical landmarks (trochanter major, lateral epicondyle, caput fibulae and lateral malleolus), defined femur and fibula lengths as well absolute knee angles. These latter were used for calibration of the goniometer.

Force applied at the lower leg was measured using a custom-made hand-held device instrumented with a bi-directional force transducer with an accuracy of 0.5 N (HBM Darmstadt, Germany). The measurement device was attached to the lower leg with a neoprene strap so that forces could be measured during both pushing as well as pulling the lower leg. The moment lever arm was measured on the line between lateral epicondyle and lateral malleolus, as the distance from the point of application of the force measurement device to the lateral epicondyle, taken as an estimate of the location of the knee axis.

Activity levels of m. biceps femoris, m. gastrocnemius medialis, m. rectus femoris and m. vastus lateralis were assessed using surface electromyograpy (EMG). Skin preparation and placement of EMG electrodes were performed according to SENIAM guidelines [29]. Force and knee angle were sampled at 100 Hz by a GSV-3USBx2-amplifier (ME-measuring systems GmbH, Germany) and Mobi system (TMSI, The Netherlands). EMG activity was sampled at 1000 Hz by a Porti or Mobi system (TMSI, The Netherlands). All signals were stored on a PC for off-line analysis.

To comfort the child, and distract him/her from the measurement a movie was played. Prior to the assessment of net knee moment-angle characteristics, the subject was asked to fully relax the leg for ten seconds, which was used to assess rest EMG (EMG-rest). As preparation for the measurement, three flexion-extension cycles from knee flexion of about 110° to knee extension of maximal 20° were performed. To avoid a knee range of motion for which the joint axes of rotation translate, knee extension between 0 and 20° was not included [30, 31]. The leg was moved only within the range that was possible without obvious EMG bursts determined by visual inspection and/or discomfort experienced by the child. After these cycles, the lower leg was pulled into a flexion position of about 110° and then slowly released till the cart stopped. From that position, the knee was extended in steps of ~5°. At each knee angle, the position was maintained for 10 seconds to allow effects of stress-relaxation. Therefore, only the last three seconds of each step were used for data analysis. After the maximal attainable extension angle was measured, the leg was slowly released and pulled towards flexion again. This procedure was repeated five times (for a typical example of three measurement cycles and selected data points, see Additional file 1, Fig. 1).

The standard method as described by Reimers was used to measure popliteal angle [12].

### Data analysis

Joint angle and force data were low-pass filtered at 1 Hz. For each knee angle, force and joint angle data were time averaged over the last three seconds of every ten second measurement interval. The net knee moment was calculated by multiplying the force measured at the force transducer by moment arm.

Since artefacts appeared to be present, below 100 Hz, all EMG data were off-line high-pass filtered at 100 Hz [32, 33] to monitor muscle activity. To obtain an envelope the filtered EMG was rectified and low-pass filtered at 5 Hz. Mean and standard deviation (SD) of the envelope data from the resting EMG were retrieved. A threshold level was set at mean + 2 SD. When mean EMG during the last three seconds of every ten second measurement interval (i.e. the interval were joint and force data was retrieved) exceeded this threshold for one of the four muscles, data corresponding to these knee joint angles were excluded from further analysis. A repetition was included for further analysis if it consisted of at least four data points, of which at least one data point lower than 0.5 Nm and at least one data point higher than 3 Nm.

Using all combinations of angles and net knee moment of a repetition, a curve was fitted by a third order polynomial function, which was retrieved based on a stepwise regression analysis using different functions.

1 $$ y=a{x}^3+b{x}^2+cx+d $$

**Fig. 2:**
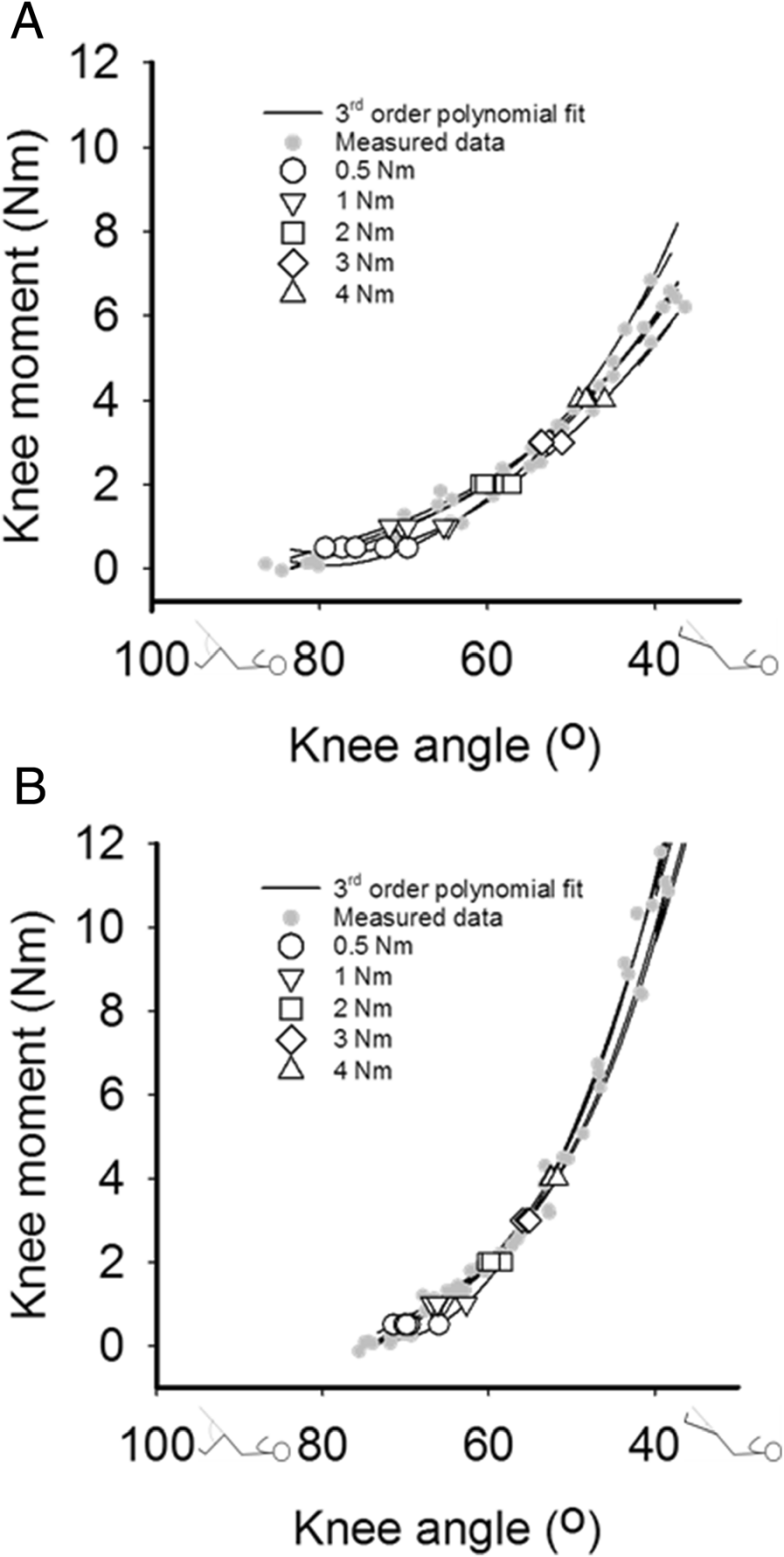
Example of five subsequently performed repetitions of knee moment-angle measurements. Typical examples of a child of the TD group (**a**) and the SCP group (**b**). Grey dots: measured data. Black lines: 3^rd^ order polynomial fit. White symbols: calculated knee angles at 0.5, 1, 2, 3 and 4 Nm

Where y represents net knee moment and x knee angle, a, b, c and d are constants determined by the fitting procedure. For comparison of repetitions, data of each repetition was fitted. As the fits of the data of the repetitions were very similar (see Additional file 2 and Additional file 3, Fig. 1 and Fig. 2), we pooled the data for the between and within-day comparison. This increased the number as well as the range of data points that can be used for fitting the data. From the fitted curves, knee angles at 0.5, 1, 2, 3 and 4 Nm and the slope at 4 Nm were derived. Higher moments were not included because these were not measured in all subjects. Knee angles at different knee moments were used for statistical analysis.

### Study design

We assessed (1) within-session reliability and measurement error from five repetitions on one day (for details see Additional file 2). Based on these data, we included three repetitions in the measurement protocol; (2) measurement error of between-day measurements from sessions on two different days; and (3) measurement error of within-day from two sessions on the same day. Between the two sessions, all devices were removed and the child walked around for 5 min.

The within-day measurement error was assessed, in addition to the between-day error, to distinguish between (1) variation as a result of possible differences in tissue properties between days and (2) variation as a result of differences in positioning of the child and alignment of the goniometer.

### Statistics

Within-session reliability was analyzed with intraclass correlation coefficients (ICC) for single measurement using variance components of subject and repetition determined by a Restricted Maximum Likelihood Estimation (RMLE) [34] (for details see Additional file 2).

The measurement errors of between-day and within-day measurements were calculated by the standard error of measurements (SEM). The SEM was determined by the standard deviation of the difference (SD_difference_) between days and SD_difference_ between sessions, respectively [34].

2 $$ \mathrm{S}\mathrm{E}\mathrm{M} = S{D}_{difference}/\sqrt{2} $$

To obtain an indication of measureable change beyond measurement error in knee angle of individual patients over time, the smallest detectable difference (SDD) was calculated. The SDD has been defined as change outside the 95 % limits of agreement [34].

3 $$ \mathrm{S}\mathrm{D}\mathrm{D} = \pm \mathrm{S}\mathrm{E}\mathrm{M} \times \sqrt{2} \times 1.96 $$

This equation is only valid in the absence of systematic difference between sessions and days. This absence was tested by Paired Sample *T*-Test for knee angles at 0.5, 1, 2, 3 and 4 Nm.

Differences between SCP and TD in anthropometric parameters, age, maximum knee angle, maximum moment and the slope at 4 Nm were tested using Independent T-Tests. Differences in net knee moment-angle characteristics between SCP and TD were tested with repeated measurement ANOVA (factors: group x moment). Correlation between knee angle at 4 Nm and the popliteal angle was calculated by the Pearson correlation coefficient (Pearson’s *r*). The level of significance was 0.05 for all statistical tests. Values are presented as means ± standard deviations (SD).

## Results

Each measurement session took about 45 min. Maximum displacement of the pelvis (12±6 mm) and upper leg (14±4 mm) did not differ between SCP and TD children. Displacement of the pelvis and upper leg between days and within days yielded very similar results. The largest displacement of pelvis and upper leg was always reached when the knee was maximally extended.

### Within-session reliability

Repeating net knee moment-angle measurements five times within one session (i.e. subject stays within the setup) resulted in similar net knee moment-angle characteristics (for typical examples, see Fig. 2).

At all net knee flexion moments tested, a single repetition yielded ICCs higher than 0.65 and SDDs ranging from 3.1° to 11.4°. Differences in ICCs between SCP and TD were similar. At all net knee flexion moments tested, averaging knee angles over three repetitions resulted in ICCs higher than 0.85 (for detailed information see Additional file 2; for all individual data, see Additional file 3, Fig. 1 and Fig. 2).

### Measurement error between days

Four subjects (3 SCP, 1 TD) could not be included on the second day due to technical or planning problems. Five SCP children and three TD children had EMG-activity higher than the EMG threshold during at least two repetitions on one of the two measurement days and were excluded from analysis of between day reliability. Therefore, between-day reliability was assessed in seven TD children and three SCP children (GMFCS I, II & III). Two to nineteen days were in between the two measurement days.

For TD and SCP children, at all moments absolute differences of mean values of the first and the second day were below 8° (Table 1). Overall values of the first and second day did not significantly differ from each other.

**Table 1:** Absolute mean differences ± standard deviation between measurement sessions and days

	TD (between-day) *n*=7	SCP (between-day) *n*=3	TD (within-day) *n*=7
Knee angle at			
0.5 Nm	7.8° ± 6.2°	5.5° ± 1.6°	6.5° ± 4.9°
1 Nm	5.8° ± 4.2°	3.8° ± 3.0°	6.7° ± 2.7°
2 Nm	5.0° ± 2.7°	4.5° ± 5.5°	6.6° ± 2.0°
3 Nm	5.5° ± 2.5°	4.4° ± 6.8°	6.1° ± 2.5°
4 Nm	6.0° ± 2.7°	4.4° ± 7.2°	5.3° ± 2.4°
Maximum Nm	5.2° ± 3.4°	6.8° ± 10.1°	5.0° ± 3.0°

*TD* typically developing children, *SCP* spastic cerebral palsy

Units are degrees

The SEMs were about 5° at knee angle corresponding to 0.5-4 Nm, which yielded SDD values from 17° (at 0.5 Nm) to 12.7° (at 4 Nm). SEM and SDD for knee angles at maximum measured knee moments were similar to those a 4 Nm (Table 2).

**Table 2:** Within- and between day measurement error: Standard error of measurement (SEM) and smallest detectable difference (SDD)

Group	Knee angle at											
	0.5 Nm		1 Nm		2 Nm		3 Nm		4 Nm		Maximum	
	SEM	SDD	SEM	SDD	SEM	SDD	SEM	SDD	SEM	SDD	SEM	SDD
TD (between-day)	6.1°	17.0°	5.0°	13.8°	4.2°	11.7°	4.3°	11.9°	4.6°	12.7°	4.6°	12.6°
TD (within-day)	6.1°	16.8°	5.4°	14.9°	5.2°	14.5°	5.0°	13.3°	4.7°	13.0°	3.9°	10.9°

*TD* typically developing children, Units of SEMs SDDs are in degree

**Fig. 3:**
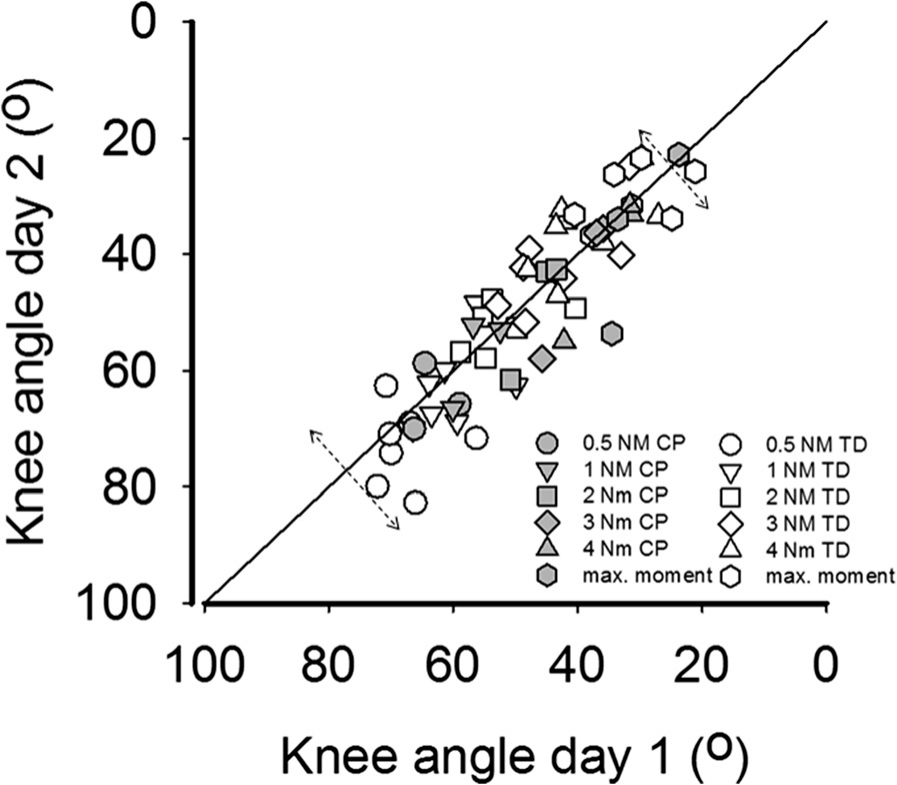
Between-day reliability of hand-held dynamometry approach to measure knee moment-angle characteristics. X-axis: knee angle at 0.5, 1, 2, 3 and 4 Nm measured at day one. Y-axis: knee angles measured at day two. Dotted arrow: between days error. TD (*n*=7) and SCP (*n*=3)

**Fig. 4:**
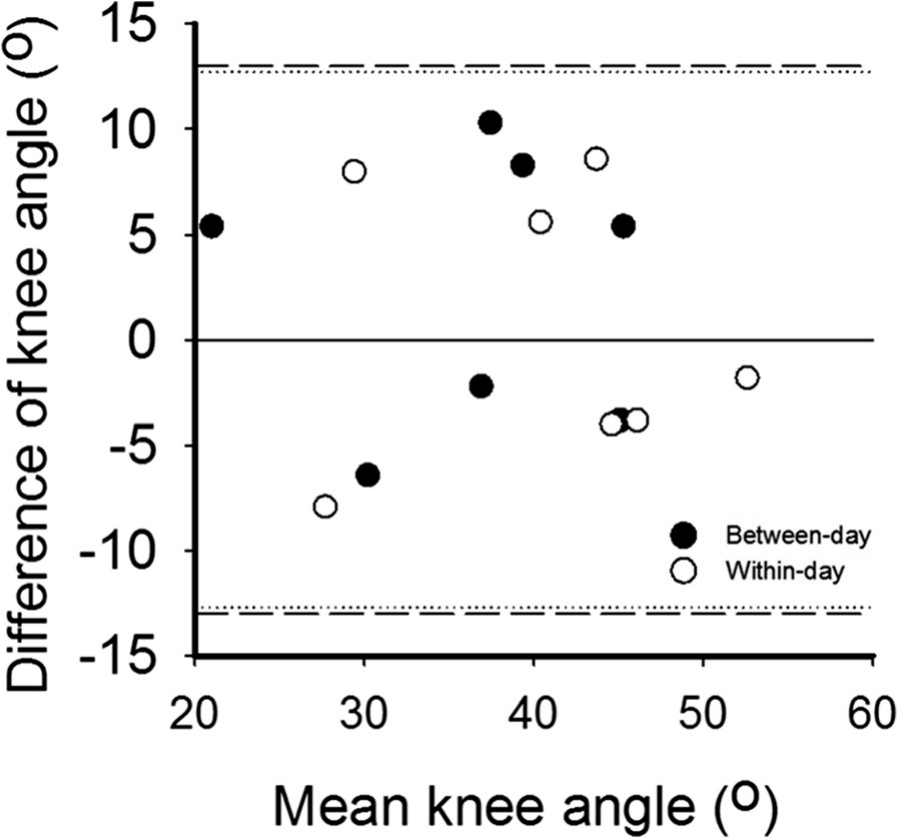
Bland and Altman plot for knee angles at 4 Nm. Net knee moment at day one and day two (between-day) (●) and session one and session two (within-day) (○). X-axis: mean values of day one and day two measurements as well as mean values of session one and session two. Y axis: difference between the knee angles (day one minus day two as well as session one minus session two). Dotted line: smallest detectable difference (SDD) of the between day reliability. Dashed line: SDD of the within-day reliability

SEMs and SDDs, were calculated for TD only, as sample size of SCP children was considered too small. For SCP children, differences in knee angles between days were within the range of those shown for TD children. Therefore, similar SEMs and SDDs for SCP children are expected (Fig. 3, Table 1; for all individual data, see Additional file 3, Fig. 3 and Fig. 4).

### Measurement error within a day

Absolute mean values of the first and the second session measured on the same day, differed 7° or less at all assessed net knee moments tested (Table 1), but differences were not systematic. SEMs and SDDs were similar to between-day measurements (Table 2).

These results show that measurement error of the net knee moment-angle characteristics within a day was similar as that of between-day measurements (see for example the comparison of data for two sessions and two days at 4 Nm, Fig. 4; for all individual data, see Additional file 3, Fig. 5).

### Correlation to popliteal angle

**Fig. 5:**
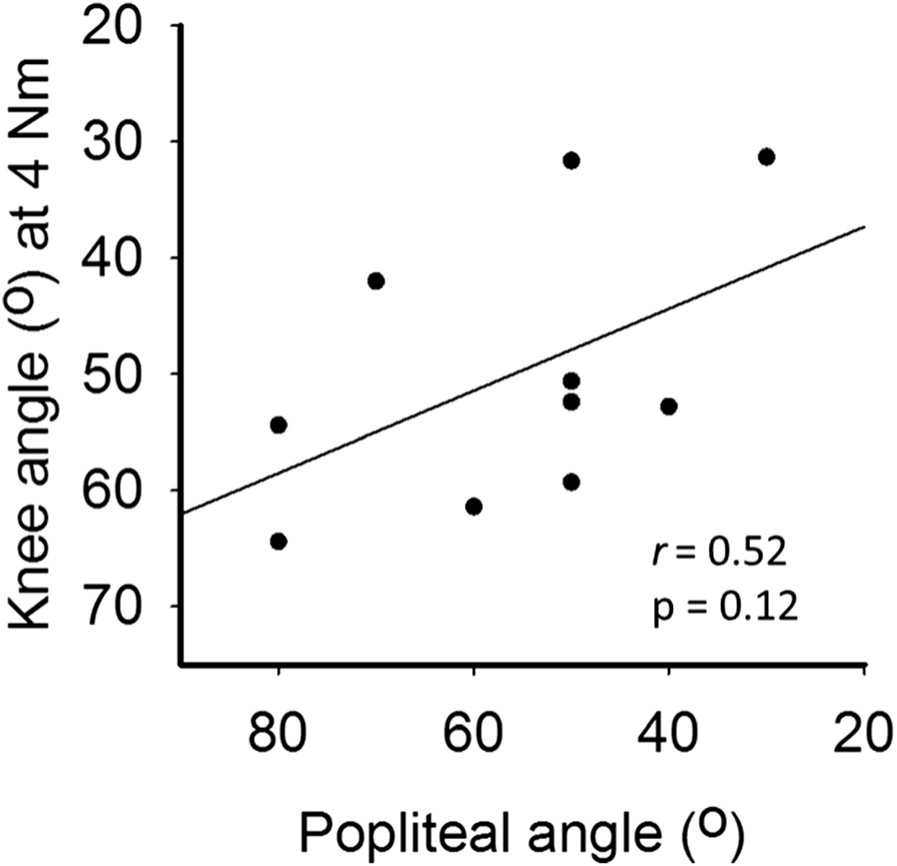
Correlation between popliteal angle and hand-held dynamometry in SCP X-axis: popliteal angle. Y-axis: knee angle at 4 Nm measured with hand-held dynamometry. (*n*=10)

For SCP children, the knee angle measured at 4 Nm net knee flexion moment was not related to the popliteal angle (Fig. 5; r=0.52; *p*=0.12).

### Difference in net knee moment-angle characteristics between SCP and TD

Eleven SPC and eleven TD were included, but one SCP child and two TD children could not perform three repetitions without significant EMG activity on at least one of the days and were excluded from comparison between the SCP and TD group.

**Fig. 6:**
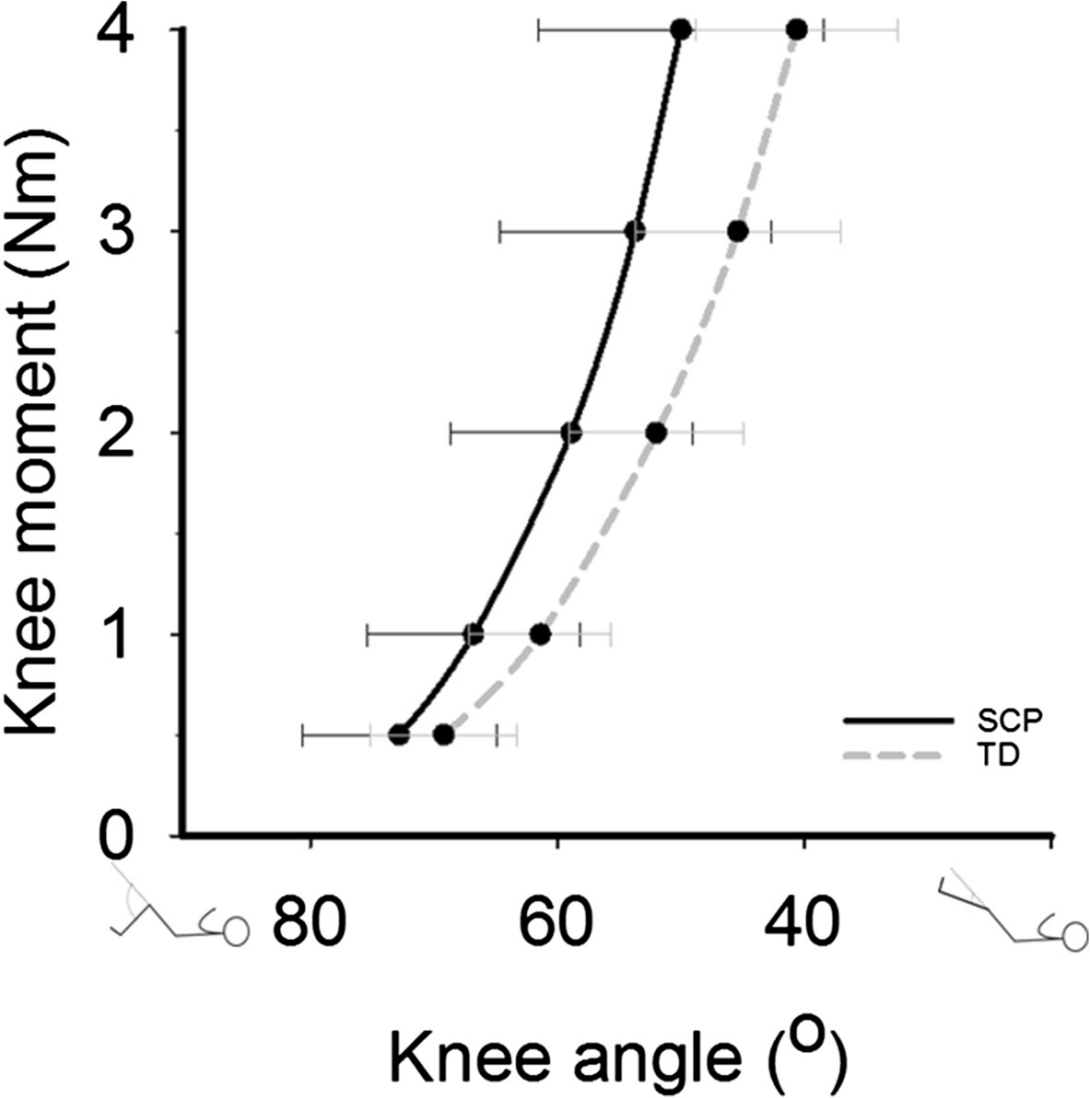
Differences of knee moment-angle characteristics of SCP and TD children. Black line: knee-moment-angle characteristics of SCP children (*n*=10). Grey line: knee-moment-angle characteristics of TD children (*n*=9). Values are means±SD

The groups did not differ in age, gender and anthropometrics (Table 3; *p*<0.05). In SCP children, knee angles measured from 0.5 to 4 Nm net knee flexion moments ranged from 72.8°±7.9° (at 0.5 Nm) to 49.7°±12.1° (at 4 Nm) and in TD from 69.2°±5.9° (at 0.5 Nm) to 40.0°±9.4° (at 4 Nm) (Table 4). Repeated measures ANOVA did not reveal an effect of groups (*p*=0.100), However, a significant interaction effect was shown between group and net knee flexion moment (*p*=0.010), which indicates that net knee moment-angle curves of SCP and TD diverged with higher net knee flexion moments (Fig. 6). The slope at 4 Nm was significantly higher in SCP children (*p*=0.017), indicating a higher increase in net knee moment as a result of knee extension. The maximum measured angle in TD children was lower (i.e. knee was more extended) than in SCP children (*p*=0.043), while the maximum measured moment did not differ (*p*=0.318) (Table 4; for all individual data, see Additional file 3, Fig. 6 and Fig. 7).

**Table 3:** Anthropometric and subject data ± standard deviation

Group	Age (years)	Gender (female/male)	Body length (cm)	Body mass (kg)	Femur length (cm)	Fibula length (cm)	GMFCS (I-III)	Popliteal angle (degree)
TD *n*=9	11.6 ± 1.7	5/4	150.2 ± 13.5	41.4 ± 10.6	34.9 ± 4.3	32.4 ± 3.7	X	X
SCP *n*=10	12.7 ± 1.7	6/4	155.9 ± 12.3	46.7 ± 11.2	36.8 ± 3.7	33.1 ± 3.4	I (4), II (3), III (3)	56 ± 16.5

*TD* typically developing children, *SCP* spastic cerebral palsy, *GMFCS* Gross Motor Function Classification System

**Table 4:** Knee angles ± standard deviation and mean differences

	SCP (*n*=10)	TD (*n*=9)	Mean difference
Knee angle at 0.5 Nm	72.8° ± 7.9°	69.2° ± 5.9°	3.6°
1 Nm	66.8° ± 8.6°	61.4° ± 5.7°	5.4°
2 Nm	58.9° ± 9.8°	52.0° ± 7.1°	6.8°
3 Nm	53.7° ± 11.0°	45.4° ± 8.3°	8.3°
4 Nm	59.7° ± 12.1°	40.6° ± 9.4°	9.8°
Maximum knee angle	42.8° ± 12.1°	32.8° ± 6.6°	9.9°^a^
Maximum knee moment	7.4 Nm ± 3.1 Nm	6.2 Nm ± 1.6 Nm	1.2 Nm
Slope at 4 Nm	0.3 ± 0.1	0.2 ± 0.1	0.1^a^

*TD* typically developing children, *SCP* spastic cerebral palsy

^a^Significantly different *p*<0.05

## Discussion

We investigated measurement error of a hand-held dynamometry approach to measure net knee moment-angle characteristics in children (TD and SCP). The results are: (1) with a SEM of about 5°, this approach allows to assess changes in individuals for knee extension angles of at least 14° between repeated measurements at positive net knee flexion moments (>0.5 Nm), (2) in SCP children the knee angle measured at 4 Nm knee moment is not related to the popliteal angle and (3) SCP children show a steeper slope of the net knee moment-angle curve at 4 Nm measured by hand-held dynamometry than TD children.

### Agreement of between day measurements of instrumented hand-held dynamometry

The evaluation of the measurement error depends on the goal of the (clinical) intervention (e.g. expected effects of treatment). Based on popliteal angle measurements, surgical lengthening of hamstring MTC is expected to result in a change of the 4 Nm knee flexion angle of more than 20° [10]. Since the SDD was 13° at 4 Nm knee flexion moment, the presented dynamometer approach allows to assess clinically meaningful changes.

Our results show that between days within one subject differences, greater than 14° in knee angles at positive net knee flexion moments (>0.5 Nm), can be assessed (i.e. differences greater than 14° are beyond the measurement error). SDD was higher for the 0.5 Nm knee angle (17°). It should be noted that when measuring a group of subjects repeatedly, SDDs are reduced by a factor square root of sample size [34]. For example, for a group of 10 subjects, the smallest difference that can detected by instrumented hand-held dynamometry is approximately 4° for net knee flexion moments between 1 and 4 Nm. Therefore, a difference of less than 5° between days can be determined in a relatively small group.

To the best of our knowledge, this is the first study that reports measurement error of net knee moment-angle characteristics while controlling for low EMG levels. The measurement of popliteal angle is to a certain extent comparable to the knee angle at 4 Nm as measured in the present study. The SDDs for between day popliteal angle measurements of (~18°) are somewhat higher [11] than those of the present study (~13°).

Our results indicate that the presented instrumented handheld dynamometer approach allows to measure clinical relevant changes in net knee flexion moment-angle characteristics at knee angles corresponding with knee moments higher than 1 Nm. Differences in knee angles that can be determined with instrumented hand-held dynamometry are 5° smaller than those determined with the commonly used popliteal angle (18°).

### Sources of error of instrumented hand-held dynamometry

Measurement errors between days may be the result of day-to-day variation in levels of muscle activity, displacement of pelvis and upper leg, mechanical muscle properties, position of the body in the setup (i.e. trunk and pelvis position; and hip and ankle angle), as well as alignment of the goniometer.

We used an EMG threshold to verify whether muscle activity of knee flexors and extensors was below the EMG threshold (see Methods). This exclusion criterion minimized the effects of EMG-activity on the moment-angle curve, but small effects of EMG activity below the threshold could occur.

Minor displacements of pelvis and upper leg were found, but these were constant during different repetitions, sessions and days. Therefore, it is not likely that these have affected the measurement error between days.

The ankle was placed in a plantar flexed position (about -20° plantar flexion for both groups) to minimize effects of gastrocnemius muscle force on knee moment. In children with SCP, zero ankle moment assessed with the knee fully extended, has been reported at ≈-10° plantar flexion [35, 36]. This implies a negligible mechanical effect of the gastrocnemius muscle at the knee for the ankle angle at which the subjects of the present study were tested.

The higher measurement error (i.e. higher SEM and SDD) at 0.5 Nm may be explained partly by friction between the cart to which the foot was attached and by the surface it was displaced on. On the toe region of the moment-angle curve, a relatively large range of knee angles at which the knee moment is near zero can be expected [37]. In those conditions, a small difference in net moment due to some friction will have a relatively large effect on the assessment of the corresponding knee angle.

The unexpected similar measurement error for the within- and between-day measurements (see Table 2) indicates that measurement error between days cannot be ascribed to differences in mechanical properties of tissues, but was rather due to variation in the performance of the measurement itself. Despite the fact that we aimed to place each child in the same position for different measurement sessions (on the same day and on different days) and to align the goniometer with respect to anatomical landmarks on the skeleton (see Methods), this procedure seemed to contribute largely to the measurement error between days, as well between sessions.

### Assessment of mechanical properties of hamstring muscles in SCP

Assessment of the popliteal angle in clinical practice aims to determine knee extension limitations due to resistance of the hamstrings muscles and joint structures as well as to estimate their contribution to crouch gait. The popliteal angle in SCP children may be increased due to an increased stiffness of the structures that span the knee joint and/or a reduction in slack length of these structures resulting in a lower knee angle at 0.5 Nm net knee moment. Therefore, interventions to decrease knee extension limitations in SCP children may be effective via distinctive mechanisms. These interventions may result either in a decrease of the slope of the net knee moment-angle curve (due to a decreased MTC stiffness) and/or a shift of the whole moment-angle curve to more extended knee angles (e.g. due to an increased MTC slack length). Such changes in anatomical structures may be distinguished by assessment of knee moment-angle characteristics, but likely not by a single point on the curve such as the popliteal angle.

To investigate to what extent differences in knee moment-angle curves are caused by differences in morphological determinants of muscle length-force characteristics (i.e. muscle belly length, fascicle length, physiological cross-sectional and pennation angle), ultrasound imaging of knee flexor and extensor muscles is deemed necessary.

The current approach (especially when combined with ultrasound measurements in future studies) provides an opportunity to assess differences in hamstring muscles properties between SCP and TD, as well as to evaluate the effects of different treatment modalities aimed at muscle lengthening (e.g. orthotics, serial casting, surgical lengthening of hamstring MTC). In addition, with our methods insight can be gained on how spasticity reduction (interventions like botulinum toxin injections and selective dorsal rhizotomy) affects mechanical and morphological properties of hamstring muscles.

The knee angle measured at 4 Nm net knee flexion moment was not related to the popliteal angle. The lack correlation may be explained by differences between popliteal angle measurements and hand-held dynamometry measurements. During popliteal angle measurements, muscle activity may affect the measured knee angle, as well as variations in hip and pelvis positions and magnitude of applied knee moment. The lower measurement error of hand-held dynamometry compared to the popliteal angle and the possibility to assess knee angles at a range of net knee moments, yield the possibility to improve the estimation of effects of hamstring muscle differences on ROM limitations and their impact on gait deviations in SCP children.

The similar knee angles at low net knee flexion moments together with a steeper slope of the curve towards higher knee moments in SCP children implies that SCP children had a reduced knee ROM within the same range of knee moments. The steeper slope of the net knee moment-knee angle curve in SCP children may be explained by increased stiffness of the MTC of the hamstring muscles, possibly due to a decreased length of muscle fascicles [22]. Furthermore, altered properties of non-muscular structures (i.e. nerves, blood vessels and their connective tissues) [38] as well as enhanced knee capsular stiffness may contribute to the steeper slope of the net knee moment-angle curve in SCP children. Altered tissue composition in muscles and tendons of SCP children may effect MTC stiffness (e.g. due to increased collagen content) [39–42]. Muscle biopsies could be used to deepen our understanding into causes of MTC stiffness in SCP.

The steeper slope of the knee moment-angle curve in SCP children may contribute to the limited ROM during gait (i.e. excursion of hamstring MTC), particularly to the restricted knee extension in terminal swing (i.e. at maximum hamstring MTC length) [8].

We assessed maximum knee moments ranging from around 4-12 Nm. In the swing phase during gait, knee flexion moments up to 0.5 Nm/kg have been reported in SCP and TD - (i.e. 20 Nm for a child of 40 kg) [43, 44]. During gait, higher moments are found as result of activation of muscles. Measurement of knee moment-angle characteristics with the current approach in SCP children may allow to distinguish the contribution to knee moments by passive mechanical tissue properties (i.e. structural alterations as shortening or stiffening of the MTC) from that neural activation (i.e. increased reflex stiffness).

In order to improve decision making for treatment of hamstring muscles, future studies are needed to combine instrumented hand-held dynamometry with ultrasound measurements and to relate these outcomes of mechanical and morphological muscle properties to joint kinematics in crouch gait. Longitudinal studies are deemed necessary to evaluate if changes in mechanical properties of muscles, tendons and ligaments will lead to a decrease in functional impairments of walking in SCP children.

### Limitations

Some limitations of the approach and the study need to be taken into account and may provide information to improve measurements of knee moment-angle characteristics in the future:

1. The current measurement protocol is quite time consuming for a standard clinical assessment (approximately 45 min) and needs specialized equipment (i.e. a custom made setup to position the child and a custom-made hand-held device for force measurement were used). However, for research purposes and for the indication of interventions with a high impact on the child (e.g. surgery) such an approach is considered justifiable.
2. In a considerable number of subjects (SCP and TD), EMG levels were higher than the set threshold (see Methods). We imposed a very strict EMG threshold to minimize anticipated effects of muscle activity on the knee moment. EMG activity seemed to increase mainly due to resistance to stretch at increased knee extension angle and that children tried to assist the extension movement. It may be possible to decrease the number of exclusions due to EMG activity with feedback to the subject on muscle activity during measurement [45], as well as by increasing the number of repetitions.
3. In our experimental design, a limited number of sources of error could be distinguished from each other. The measurement error is a sum of different errors that can be the result of (a) differences in mechanical tissue properties, (b) differences in body position in the setup (i.e. differences in pelvis position and joint angles at hip and ankle), (c) different placement of the goniometer on the skin, (d) instrumentation errors or (e) errors made by de examiner. To obtain insight in the contribution of these sources of error to the overall measurement error for the introduced approach would require an extended study design.

## Conclusions

Instrumented knee hand-held dynamometry as presented in this study allows to assess clinically relevant changes in net knee moment-angle characteristics in TD and SCP children. Valuable information about stiffness of the hamstring muscles and other structures spanning the knee joint can be obtained. This information can be useful to quantify functional changes in SCP after clinical interventions and to study mechanisms underlying the outcomes of these interventions.

## Additional files

Additional file 1:
**Selection of data points from raw measurement data.**
Additional file 2:
**Within session reliability of measurements of knee moment-angle characteristics.**
Additional file 3:
**All individual measurement data with appertaining fit and extracted data points.**

## Abbreviations

SCP: Spastic cerebral palsy
TD: Typically developing
MTC: Muscle-tendon complex
ROM: Range of motion
GMFCS: Gross Motor Function Classification System
EMG: Electromyogram
SD: Standard deviation
ICC: Intraclass correlation coefficient
SEM: Standard error of measurement
SDD: Smallest detectable difference
