# Supplementary material for: Assessment of net knee moment-angle characteristics by instrumented hand-held dynamometry in children with spastic cerebral palsy and typically developing children
Source: J Neuroeng Rehabil. 2015 Aug 15;12:67. doi: 10.1186/s12984-015-0056-y (PMC4536590; doi:10.1186/s12984-015-0056-y)
Supplement: Additional file 1: — Selection of data points from raw measurement data. [file 12984_2015_56_MOESM1_ESM.pdf]

Additional file 1

Selection of data points from raw measurement data

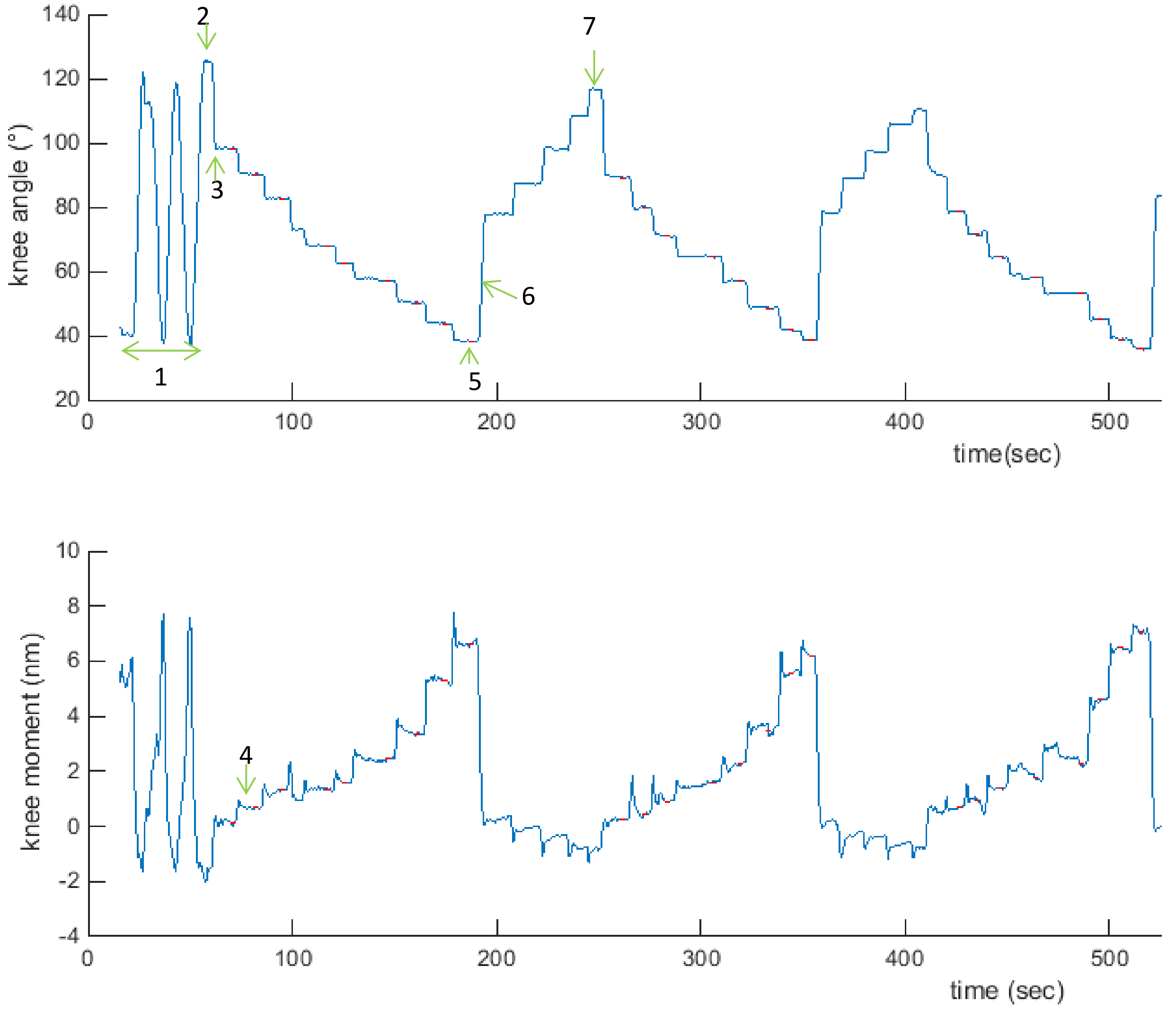

Fig. 1. **Typical example of recorded angle and moment for three cycles of flexion and extension movement:** As preparation for the measurement, three flexion-extension cycles from knee flexion of about 110° to knee extension of maximal 20° were performed (1). The leg was moved only within the range that was possible without obvious EMG bursts determined by visual inspection and/or discomfort experienced by the child. After these cycles, the lower leg was pulled into a flexion position of about 110° (2) and then slowly released till the cart stopped (3). From that position, the knee was extended in steps of ~5°. At each knee angle, the position was maintained for 10 seconds to allow effects of stress-relaxation (e.g. 4). Therefore, only the last three seconds of each step were used for data analysis (red bar). After the maximal attainable extension angle (5) was measured, the leg was slowly released (6) and pulled in small steps towards flexion again (7). In this example three repetitions are shown.
