# Supplementary material for: Assessment of net knee moment-angle characteristics by instrumented hand-held dynamometry in children with spastic cerebral palsy and typically developing children
Source: J Neuroeng Rehabil. 2015 Aug 15;12:67. doi: 10.1186/s12984-015-0056-y (PMC4536590; doi:10.1186/s12984-015-0056-y)
Supplement: Additional file 2: — Within session reliability of measurements of knee moment-angle characteristics. [file 12984_2015_56_MOESM2_ESM.pdf]

Additional file 2

Within session reliability of measurements of knee moment-angle characteristics

We assessed within-session reliability from five repetitions. Within a session the subject stayed within the setup. 11 children with SCP and 11 typically develop children were included**.** Six TD and six SCP were excluded for analyses due to beyond threshold EMG activity at least during one repetition.

Statistics

Variance components of the factors subject (σ^2_p), repetition (σ^2_r) and the residual error (σ^2_e) were determined using Restricted Maximum Likelihood Estimation (RMLE) [1]. Subject variance represents the variance between subjects (i.e. variance of interest) and the repetition variance component represents the error variance, caused by repetition. The residual error is the sum of the interaction subject x repetition and a random error (e.g. caused by examiner or instrumentation).

Reliability was assessed by the intraclass correlation coefficients (ICC) for single measurements and the smallest detectable difference (SDD). Since we were interested in absolute agreement of measurements we used to calculate the ICC from the variance components [1, 2]:

$$\text{ICC} = \frac{variance\ of\ interest}{variance\ of\ interest + error\ variances} \quad \text{(Equation 1)}$$

ICC values range between 0 and 1. An ICC towards 1 indicates that error variance is negligible compared to inter subject variance and that subjects can be well distinguished from each other [1]. To obtain an indication of measureable true change in knee angle of individual patients over time, we calculated from the error variance the standard error of measurements (SEM) and smallest detectable difference (SDD) [2]. By definition, 95% of the differences between

measurements lies within the limits of agreement. The SDD has been defined as difference outside the limits of agreement [1]:

$$\text{SEM} = \sqrt{\sigma^2{}_r + \sigma^2{}_e} \text{ (Equation 2)},$$

$$\text{SDD} = \pm\, \text{SEM} \times \sqrt{2} \times 1.96 \text{ (Equation 3)}$$

To determine the increase in reliability with number of repetitions, we divided the variance components of repetition and error variance by number of repetitions [1].

Results

Repeating net knee moment-angle measurements five times within one session (i.e. subject stays within the setup) resulted in similar net knee moment-angle characteristics (for typical examples, see Fig.2 main manuscript and Additional file 3, Fig. 1 and Fig 2). The estimates of the variance components revealed that most of the variance in knee angle at particular net knee flexion moments was explained by subject (i.e. variance of interest), with maximum values for subject variance at 4 Nm. Residual error variance was the main source of error. Values of residual error variance were similar for moments ranging from 2-4 Nm and substantially higher at 0.5 and 1 Nm. Estimates of variance components are reported in Table 1.

At all net knee flexion moments tested, a single repetition yielded ICCs higher than 0.65 and SDDs ranging from 3.1^{o} to 11.4^{o}. Differences in ICCs between SCP and TD were similar (Table 2).

Conclusion

At all net knee flexion moments tested, averaging knee angles over three repetitions resulted in ICCs higher than 0.85. These data indicate that three repetitions are sufficient to assess measurement error of net knee moment-angle characteristics between days and sessions.

Tables

Table 1 Estimates of variance components (σ^2) of subject, repetition and the residual error variance for the knee angle at different knee moments:

Group	Age (years) GMFCS	Sources of Variance	Degree of Freedom	σ^2 Knee angle at				
				0.5 Nm	1 Nm	2 Nm	3 Nm	4 Nm
TD	10.9±1.3 years	Subject	4	98.9	84.3	97.4	109.3	119.1
		Repetition	4	5.6	3.0	0.0	0.0	0.0
		Residual	20	8.4	4.2	1.9	2.1	2.0
SCP	13.9±0.6 years, GMFCS:I-II	Subject	44	32.2	42.0	56.0	68.1	79.7
		Repetition	20	3.2	0.1	0.3	0.2	0.1
		Residual		13.6	11.4	3.2	1.6	1.1

TD= typically developing children, SCP=spastic cerebral palsy, GMFCS= Gross Motor Function Classification System . Units for σ^2 knee angle at 0-4 Nm are degree^2.

Tabel 2 Intraclass correlation coefficient (ICC) and smallest detectable difference (SDD) for 1-5 repetitions.

Group	rep	Knee angle at									
		0.5 Nm		1 Nm		2 Nm		3 Nm		4 Nm	
		ICC	SDD	ICC	SDD	ICC	SDD	ICC	SDD	ICC	SDD
TD n=5	1	0.88	10.4	0.92	7.4	0.98	3.8	0.98	4.0	0.98	4.0
	2	0.93	7.3	0.96	5.3	0.99	2.7	0.99	2.8	0.99	2.8
	**3***	**0.95**	**6.0**	**0.97**	**4.3**	**0.99**	**2.2**	**0.99**	**2.3**	**0.99**	**2.3**
	4	0.97	5.2	0.98	3.7	1.00	1.9	1.00	2.0	1.00	2.0
	5	0.97	4.6	0.98	3.3	1.00	1.7	1.00	1.8	1.00	1.8
SCP n=5	1	0.65	11.4	0.79	9.4	0.94	5.2	0.97	3.8	0.98	3.1
	2	0.79	8.1	0.88	6.7	0.97	3.7	0.99	2.7	0.99	2.2
	**3***	**0.85**	**6.6**	**0.92**	**5.4**	**0.98**	**3.0**	**0.99**	**1.8**	**0.99**	**1.8**
	4	0.88	5.7	0.94	4.7	0.98	2.6	0.99	1.9	1.00	1.6
	5	0.90	5.1	0.95	4.2	0.98	2.3	0.99	1.7	1.00	1.4

TD= typically developing children, SCP=spastic cerebral palsy ICC is dimensionless (0-1). Units of SDDs are in degree. *Averaging over three repetitions is sufficient to assess the net knee moment-angle curve, with more repetitions SDDs only slightly improve.

References

1. Vet HCW, Terwee CB, L.B. M, Knol DL. Measurement in Medicine. Cambridge University Press; 2011.
2. Roebroeck ME, Harlaar J, Lankhorst GJ. The application of generalizability theory to reliability assessment: an illustration using isometric force measurements. Phys Ther. 1993;73(6):386-95; discussion 96-401.
