# Supplementary material for: Assessment of net knee moment-angle characteristics by instrumented hand-held dynamometry in children with spastic cerebral palsy and typically developing children
Source: J Neuroeng Rehabil. 2015 Aug 15;12:67. doi: 10.1186/s12984-015-0056-y (PMC4536590; doi:10.1186/s12984-015-0056-y)
Supplement: Additional file 3: — All individual measurement data with appertaining fit and extracted data points. [file 12984_2015_56_MOESM3_ESM.pdf]

Additional file 3

All individual measurement data with appertaining fit and extracted data points

In Fig. 1.and Fig. 2 all individual data of the within session comparison (i.e. five repetitions) of five children with spastic cerebral palsy and typically developing children are presented.

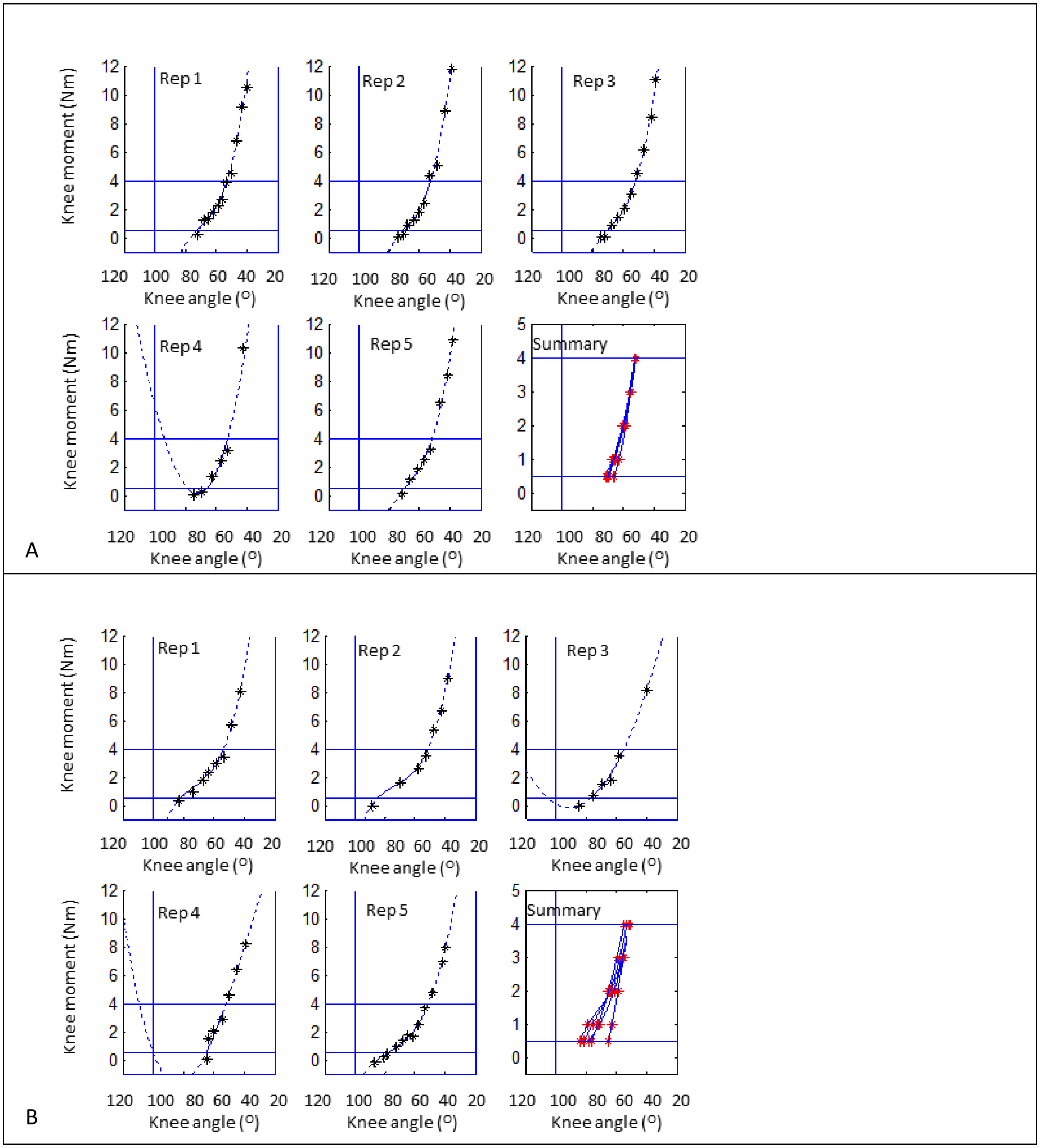

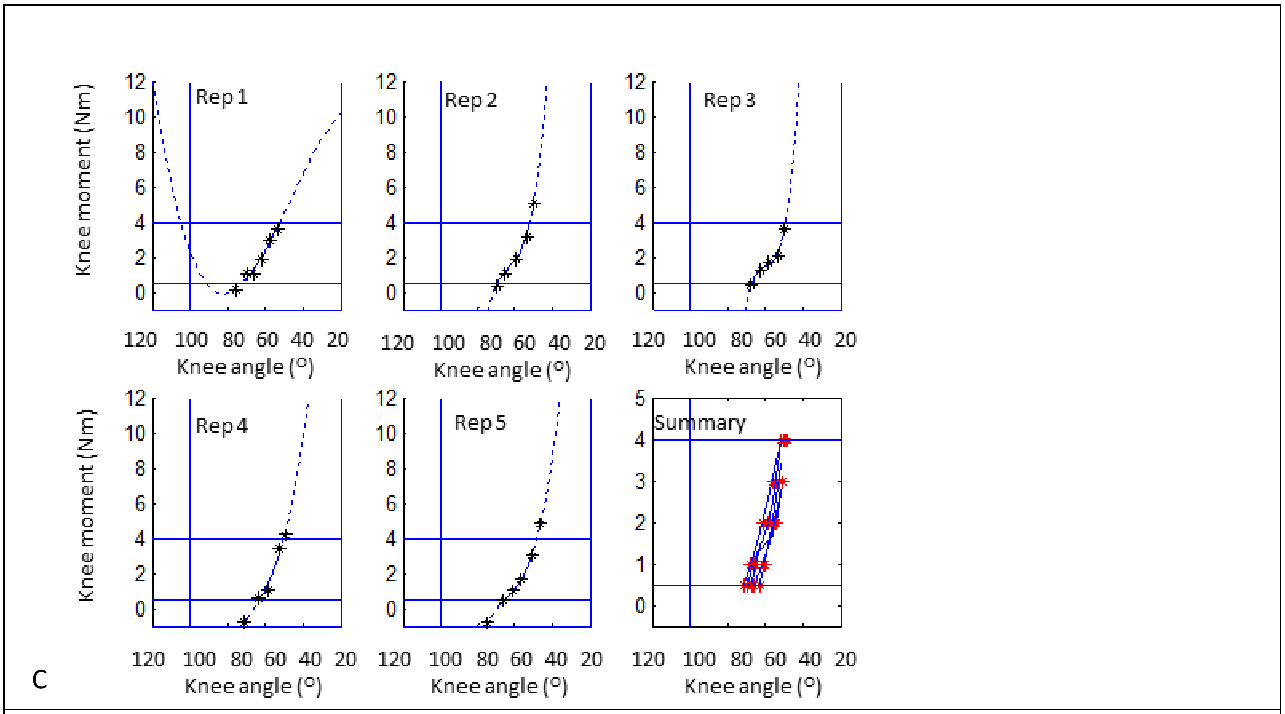
Rep 1
Rep 2
Rep 3
Rep 4
Rep 5
Summary
Knee moment (Nm)
Knee angle (°)
C

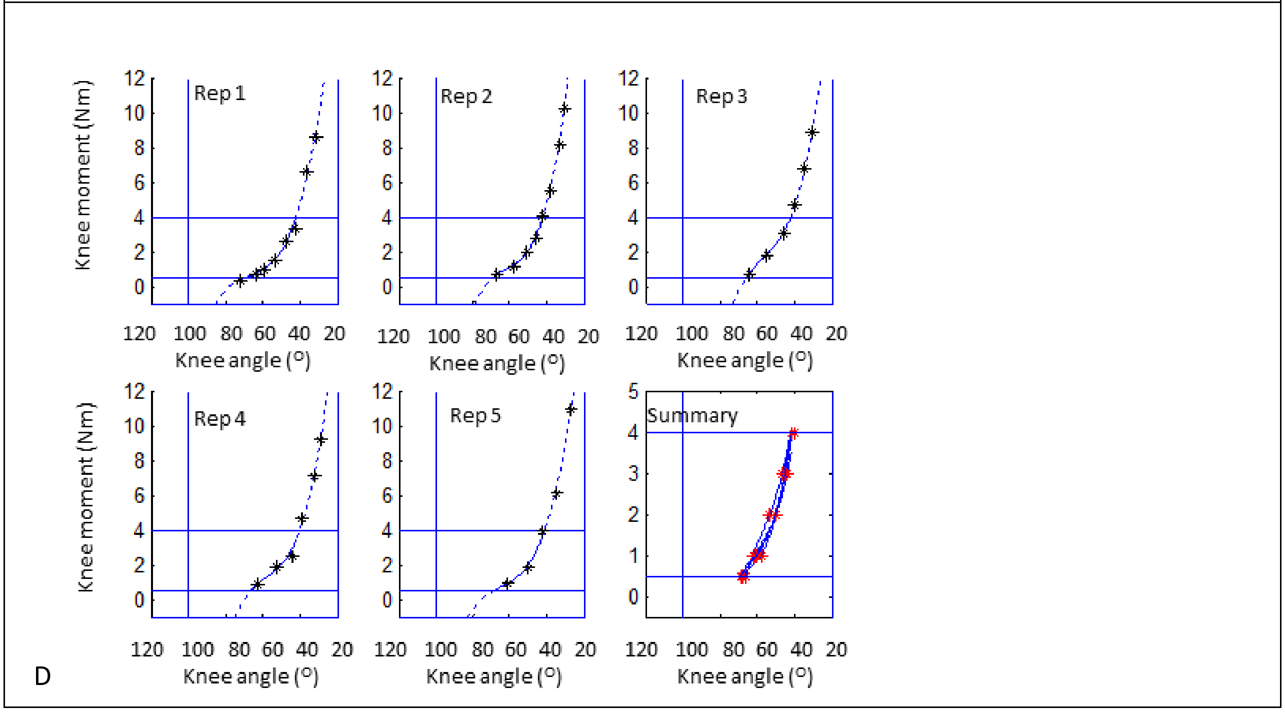
Rep 1
Rep 2
Rep 3
Rep 4
Rep 5
Summary
Knee moment (Nm)
Knee angle (°)
D

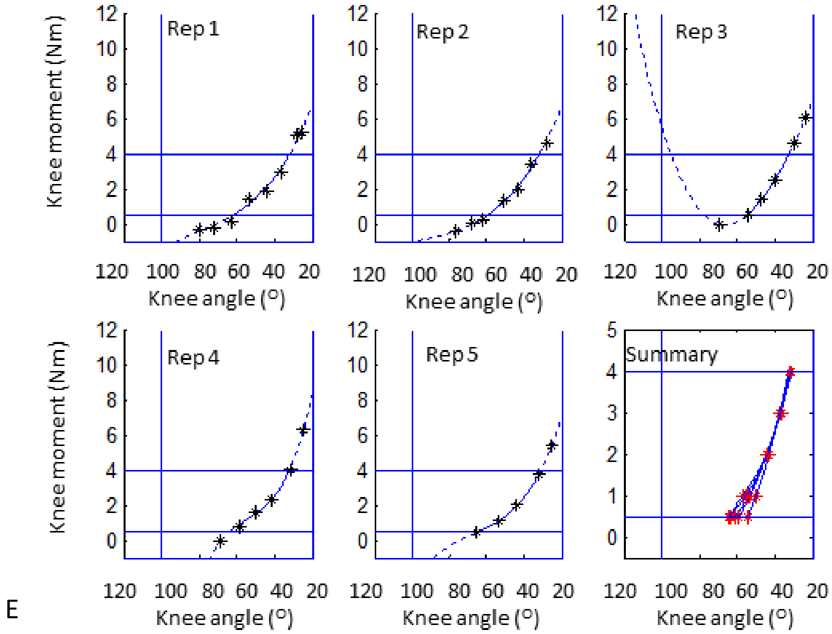

Fig. 1. **Individual data of five children with spastic cerebral palsy - five repetitions and summary plot (A-E):** Black *=individual measured data points after exclusion of data points beyond EMG-threshold. Blue dashed line=fitted function (i.e. 3rd order polynomial function). Blue solid line=fitted line within the range used for comparison (i.e. knee angles corresponding to 0.5-4 Nm). Red *= derived estimates of the fit for statistical comparison at 0.5, 1, 2, 3 and 4 Nm.

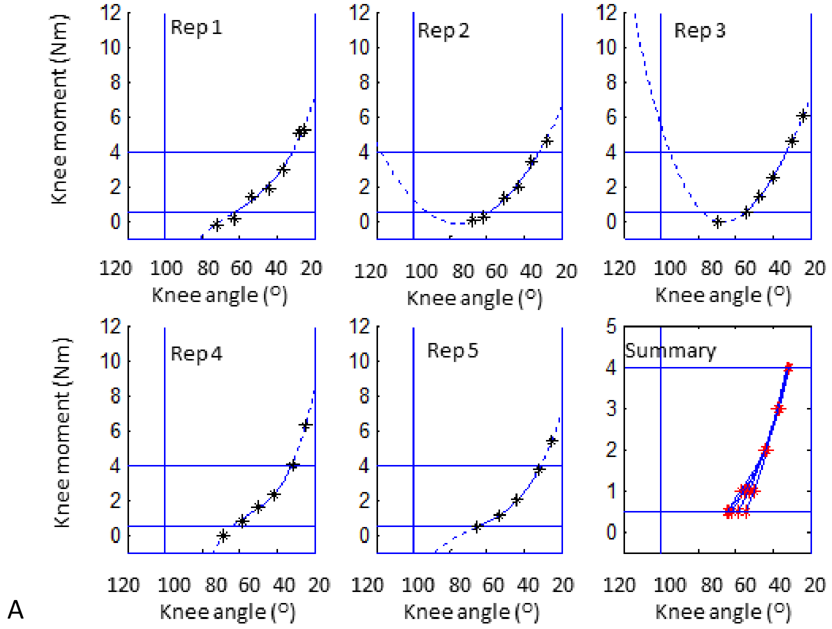

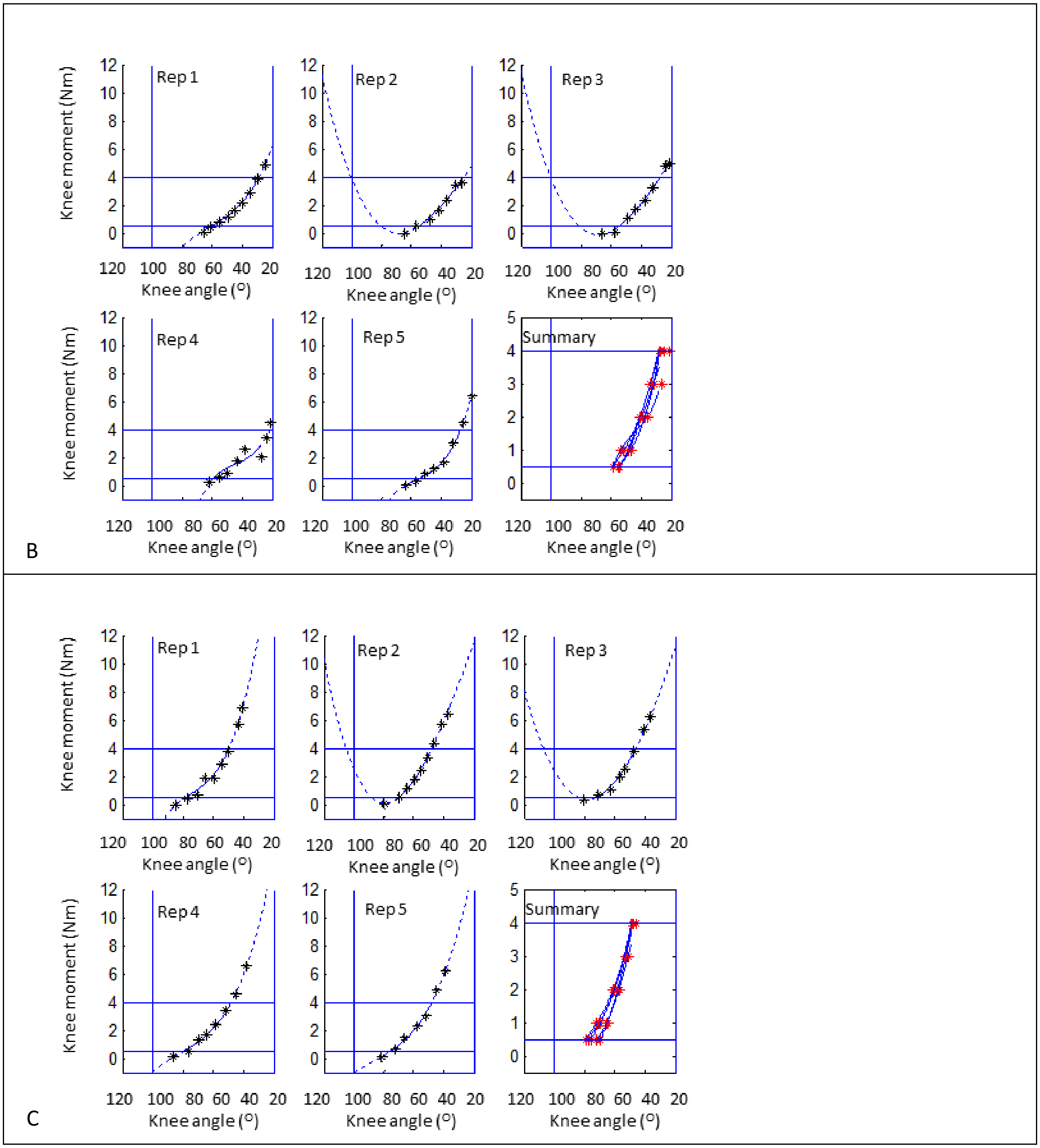
B
Rep 1
Rep 2
Rep 3
Rep 4
Rep 5
Summary
Knee moment (Nm)
Knee angle (°)
C
Rep 1
Rep 2
Rep 3
Rep 4
Rep 5
Summary
Knee moment (Nm)
Knee angle (°)

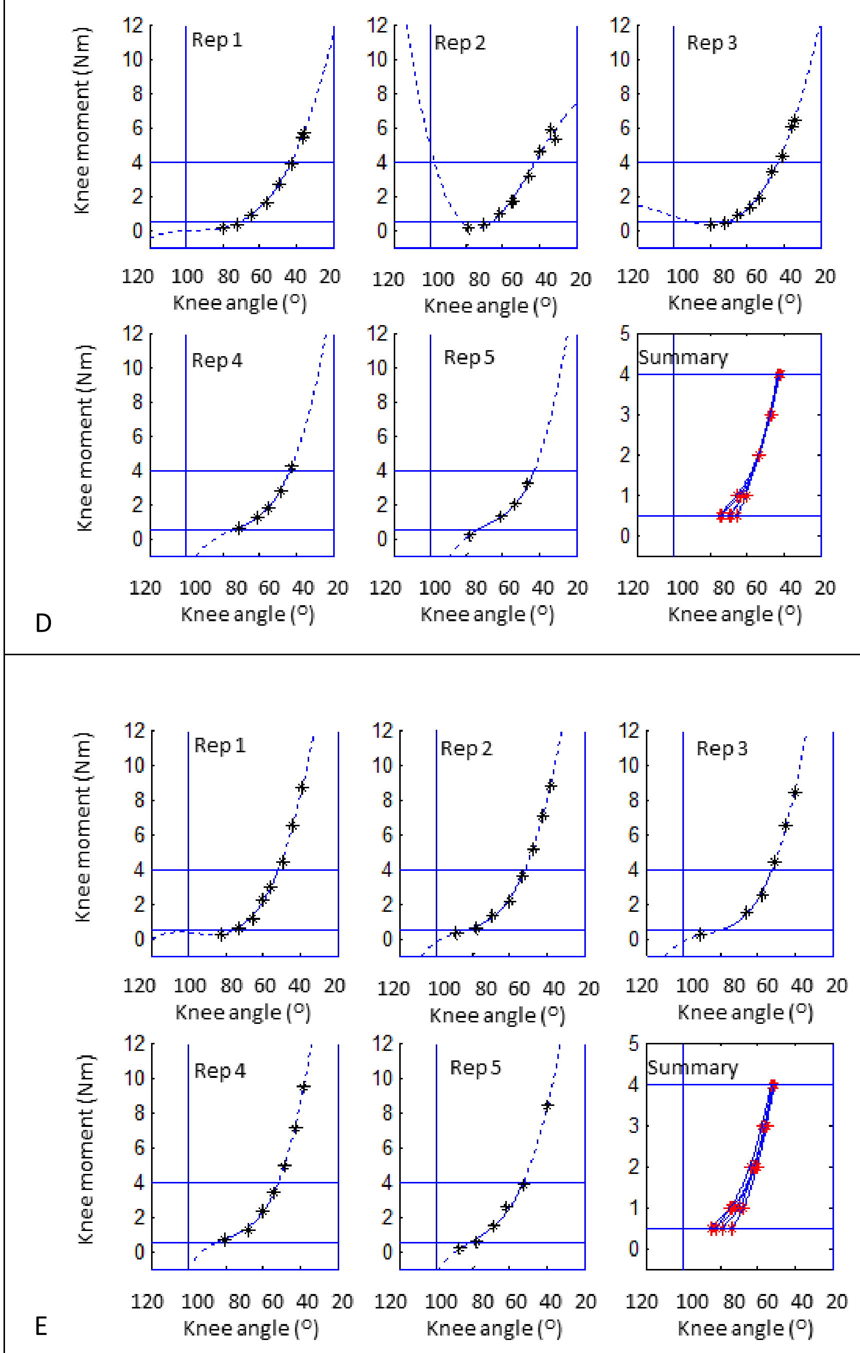

Fig. 2. **Individual data of five typically developing children - five repetitions and summary plot (A-E):** Black *=individual measured data points after exclusion of data points beyond EMG-threshold. Blue dashed line=fitted function (i.e. 3rd order polynomial function). Blue solid line=fitted line within the range used for comparison (i.e. knee angles corresponding to 0.5-4 Nm). Red *= derived estimates of the fit for statistical comparison at 0.5, 1, 2, 3 and 4 Nm.

In Fig. 3. and Fig. 4 all individual data of between day comparison of three children with spastic cerebral palsy and seven typically developing children are presented.

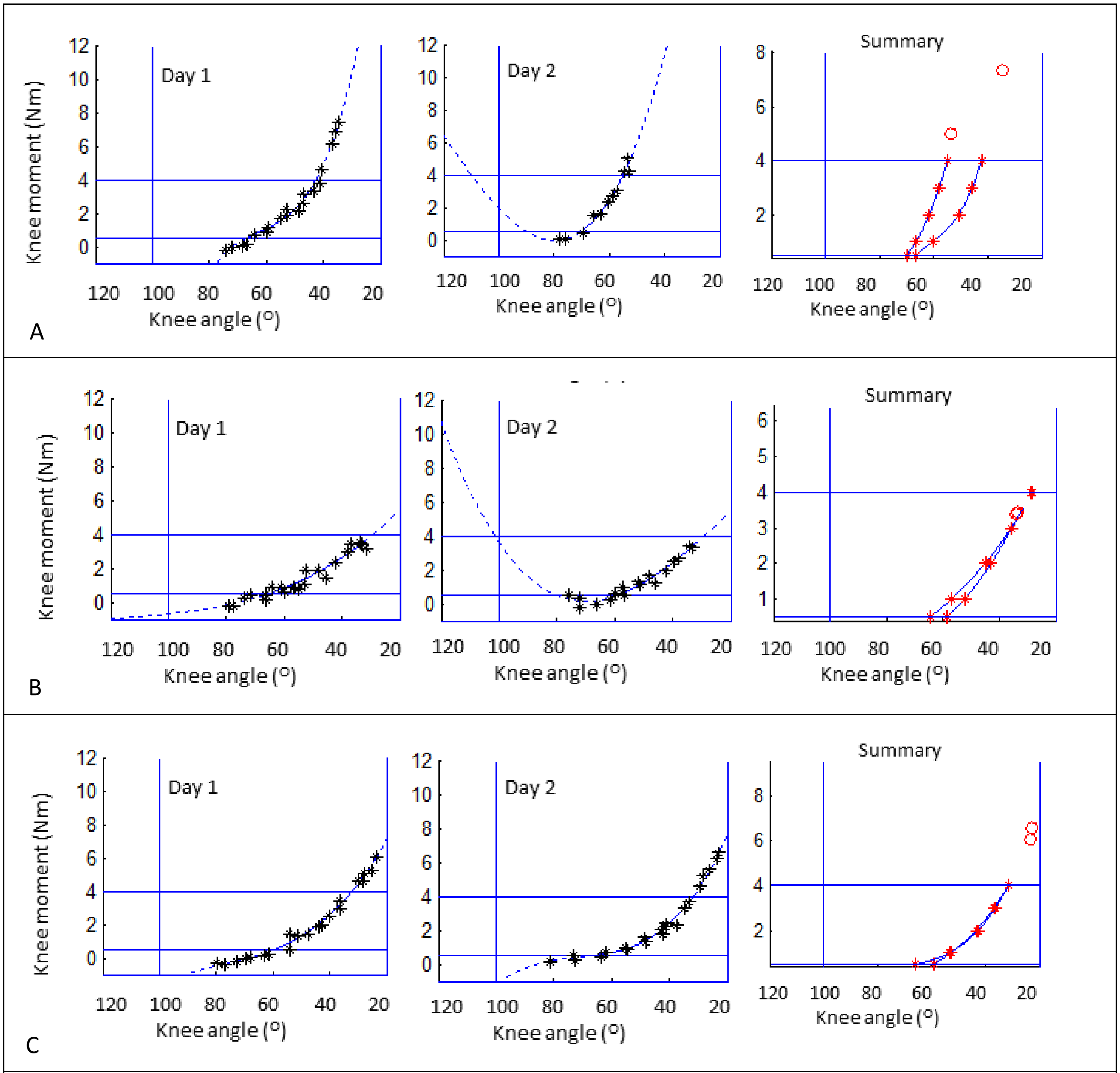

Fig. 3. **Individual data of three children with spastic cerebral palsy – pooled data of three repetitions for both days and summary plot (A-C):** Black *=individual measured data points after exclusion of data points beyond EMG-threshold. Blue dashed line=fitted function (i.e. 3rd order polynomial function). Blue solid line=fitted line within the range used for comparison (i.e. knee angles corresponding to 0.5-4 Nm). Red *= derived estimates of the fit for statistical comparison at 0.5, 1, 2, 3 and 4 Nm. Red °= maximum measured angle.

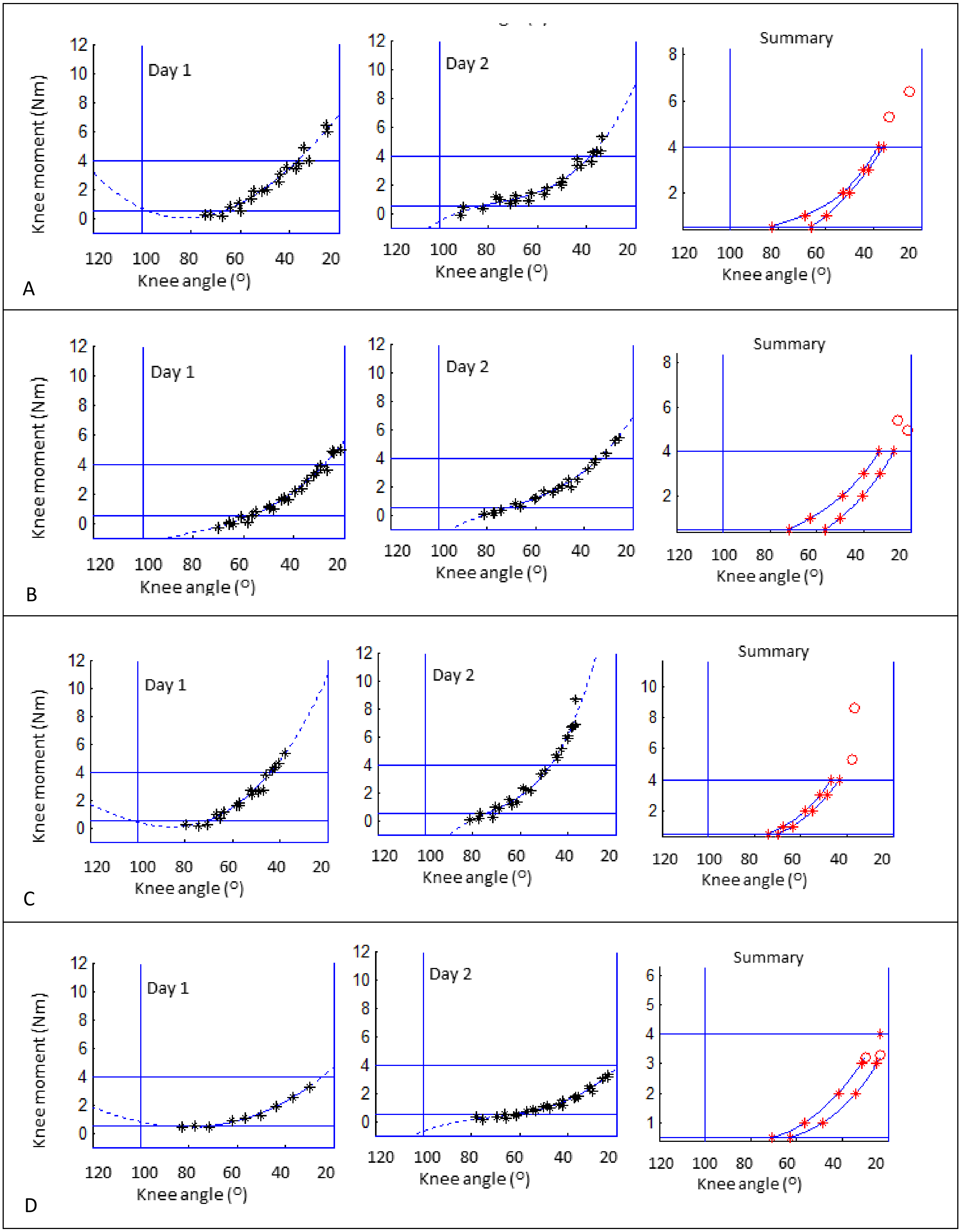
Knee moment (Nm)
Day 1
Day 2
Summary
Knee angle (°)
A
B
C
D

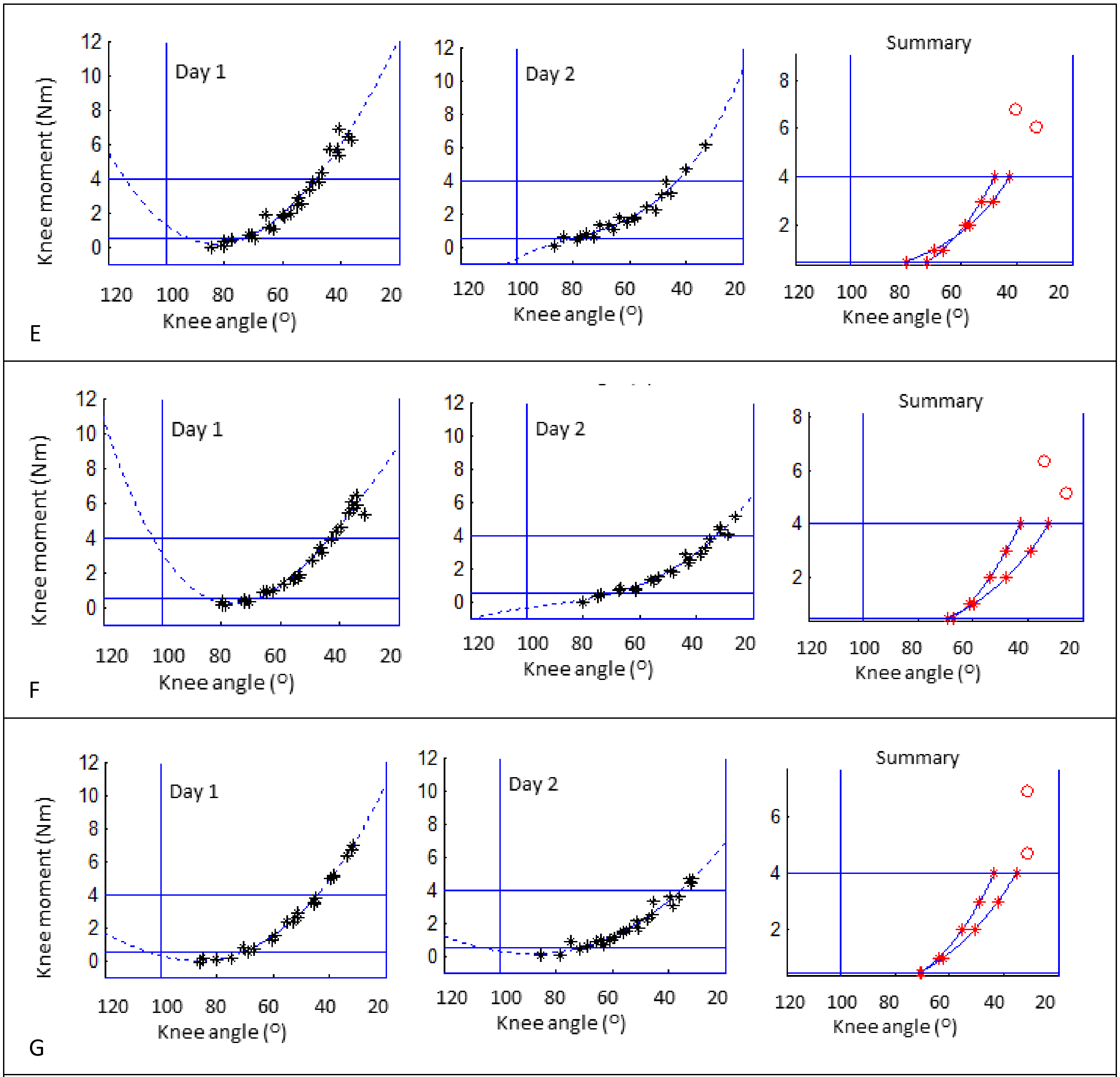

Fig. 4. **Individual data of seven typically developing children– pooled data of three repetitions on two days and summary plot (A-G):** Black *=individual measured data points after exclusion of data points beyond EMG-threshold. Blue dashed line=fitted function (i.e. 3rd order polynomial function). Blue solid line=fitted line within the range used for comparison (i.e. knee angles corresponding to 0.5-4 Nm). Red *= derived estimates of the fit for statistical comparison at 0.5, 1, 2, 3 and 4 Nm. Red °= maximum measured angle.

In Fig. 5.all individual data of within day comparison of seven typically developing children are presented.

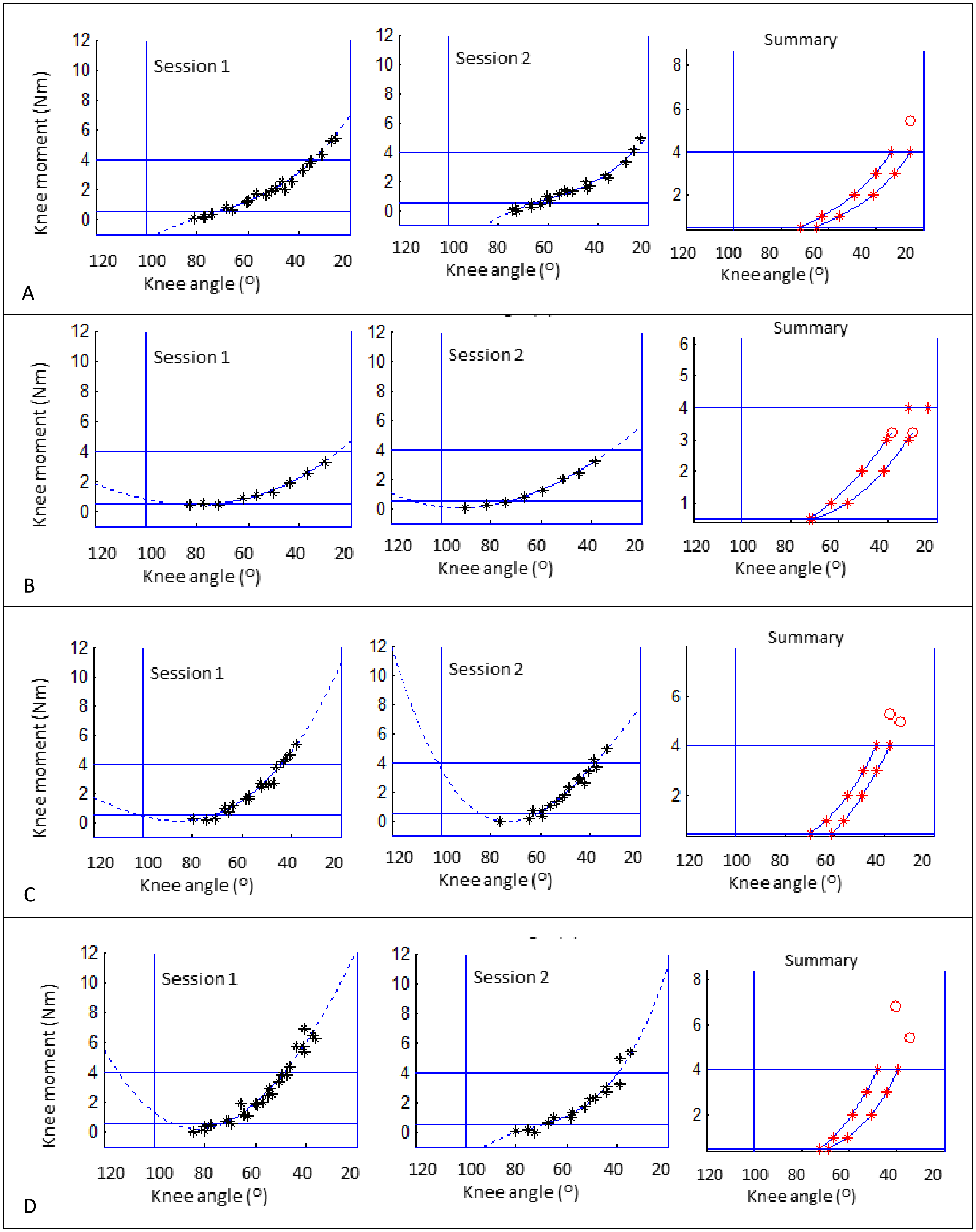

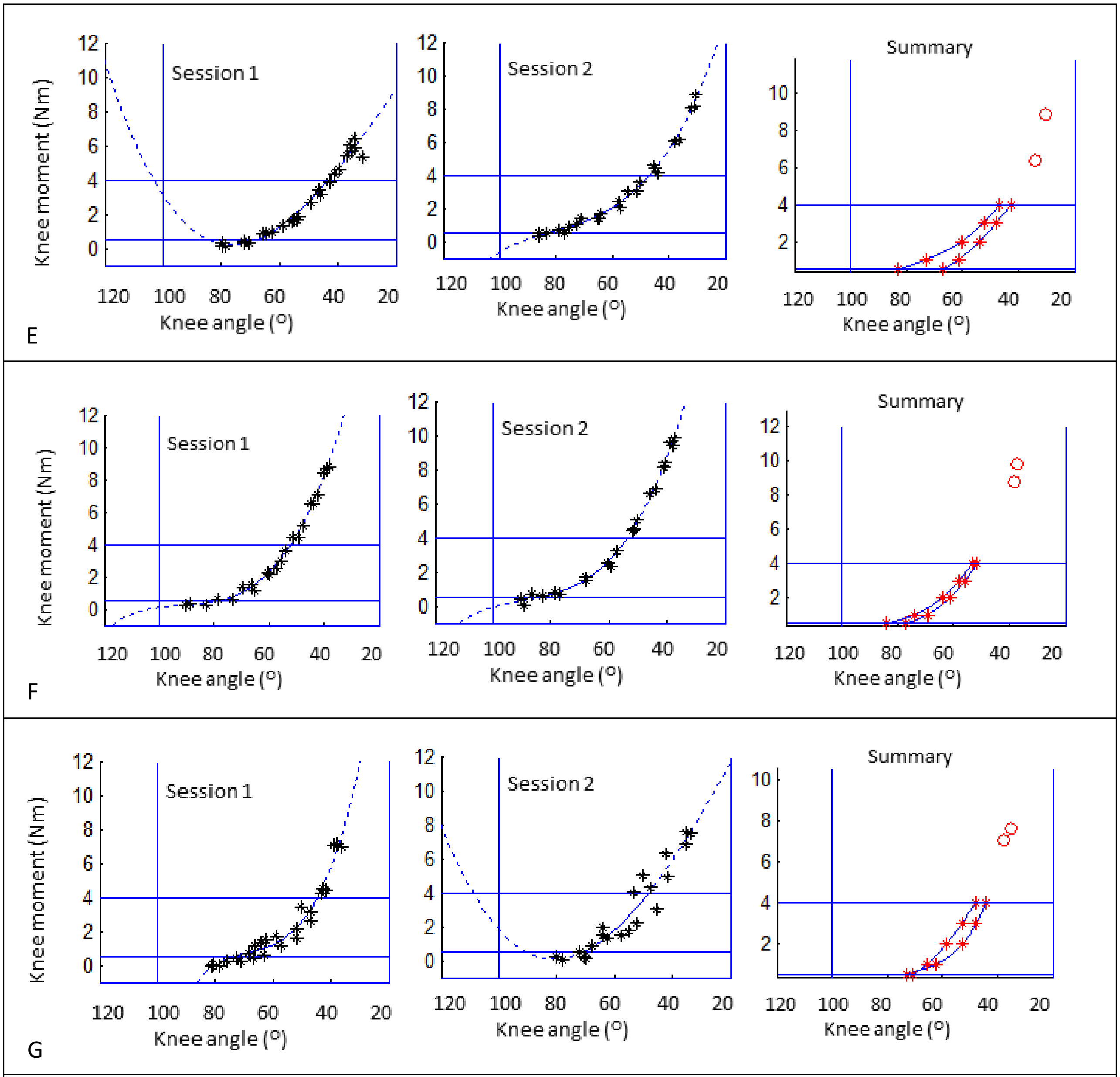

Fig. 5. **Individual data of seven typically developing children– pooled data of three repetitions for two sessions and summary plot (A-G):** Black *=individual measured data points after exclusion of data points beyond EMG-threshold. Blue dashed line=fitted function (i.e. 3rd order polynomial function). Blue solid line=fitted line within the range used for comparison (i.e. knee angles corresponding to 0.5-4 Nm). Red *= derived estimates of the fit for statistical comparison at 0.5, 1, 2, 3 and 4 Nm. Red °= maximum measured angle.

In Fig. 6 and Fig. 7 all individual data for the group comparison between children with cerebral palsy (10 children) and typically developing children (nine children) are presented.

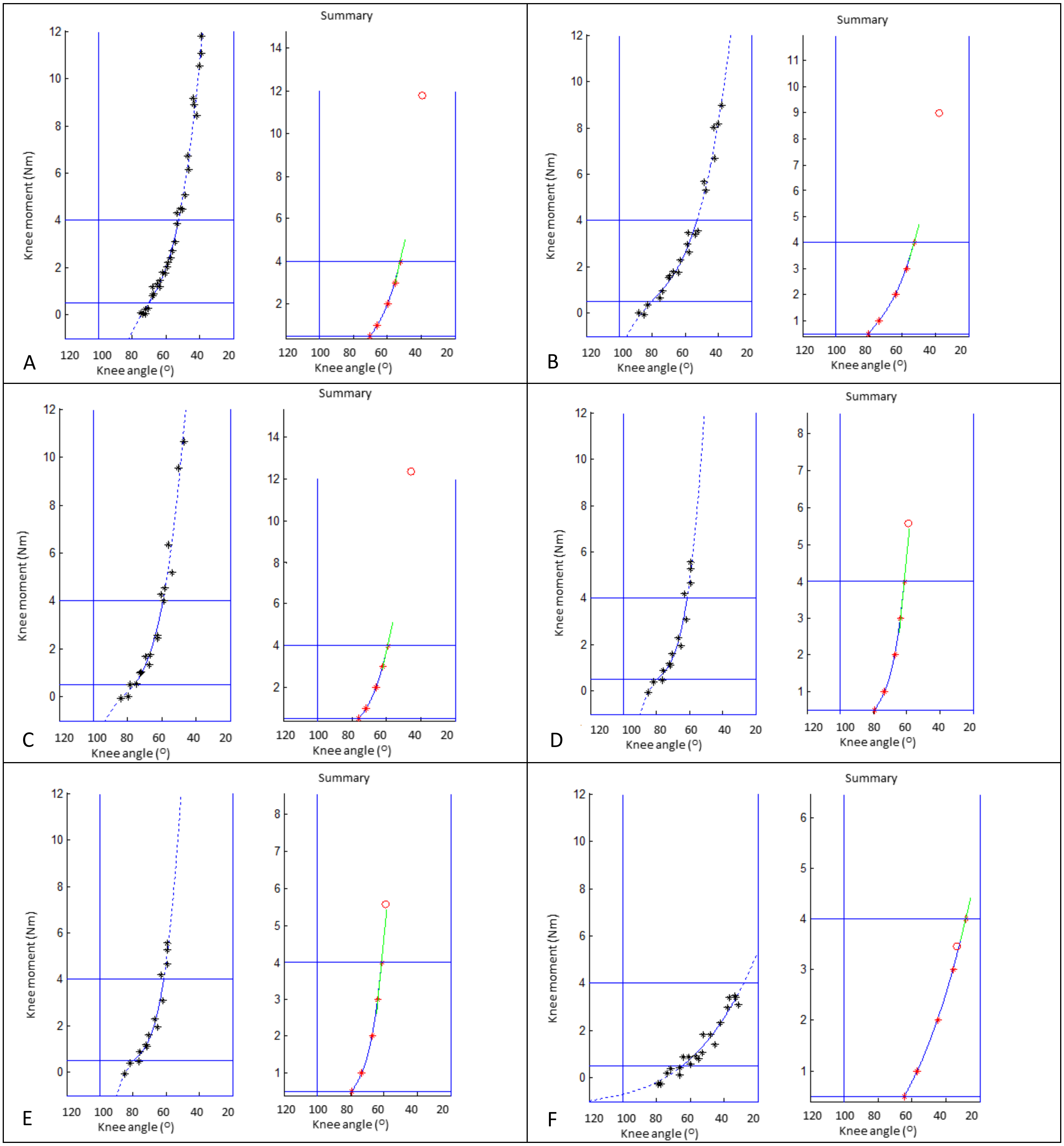

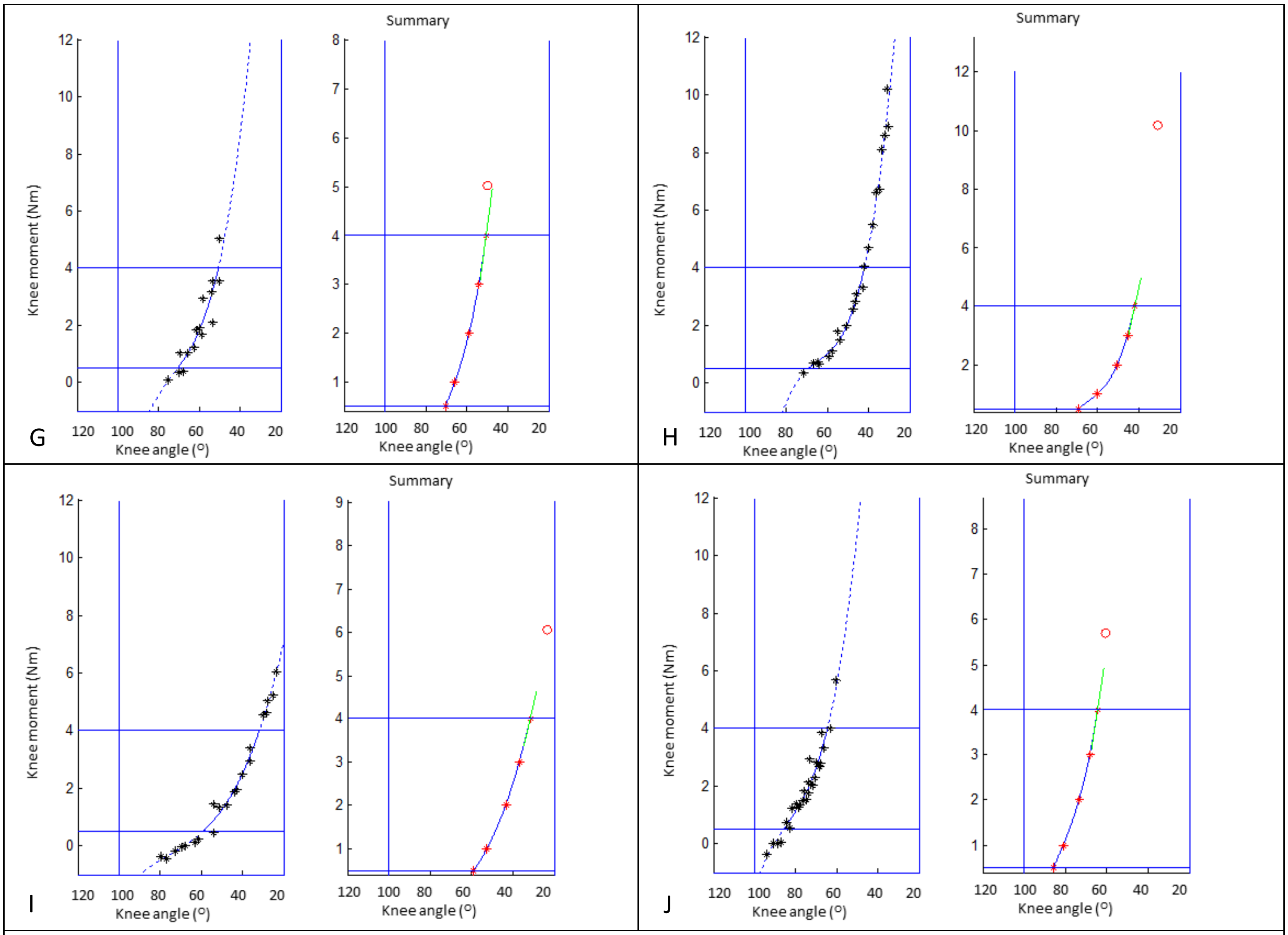

Fig. 6. **Individual data of ten children with spastic cerebral palsy – pooled data of three repetitions and summary plot (A-J):** Black *=individual measured data points after exclusion of data points beyond EMG-threshold. Blue dashed line=fitted function (i.e. 3rd order polynomial function). Blue solid line=fitted line within the range used for comparison (i.e. knee angles corresponding to 0.5-4 Nm). Red *= derived estimates of the fit for statistical comparison at 0.5, 1, 2, 3 and 4 Nm. Red °= maximum measured angle. Green line = tangent line at 4 Nm (slope at 4 Nm is derived for comparison).

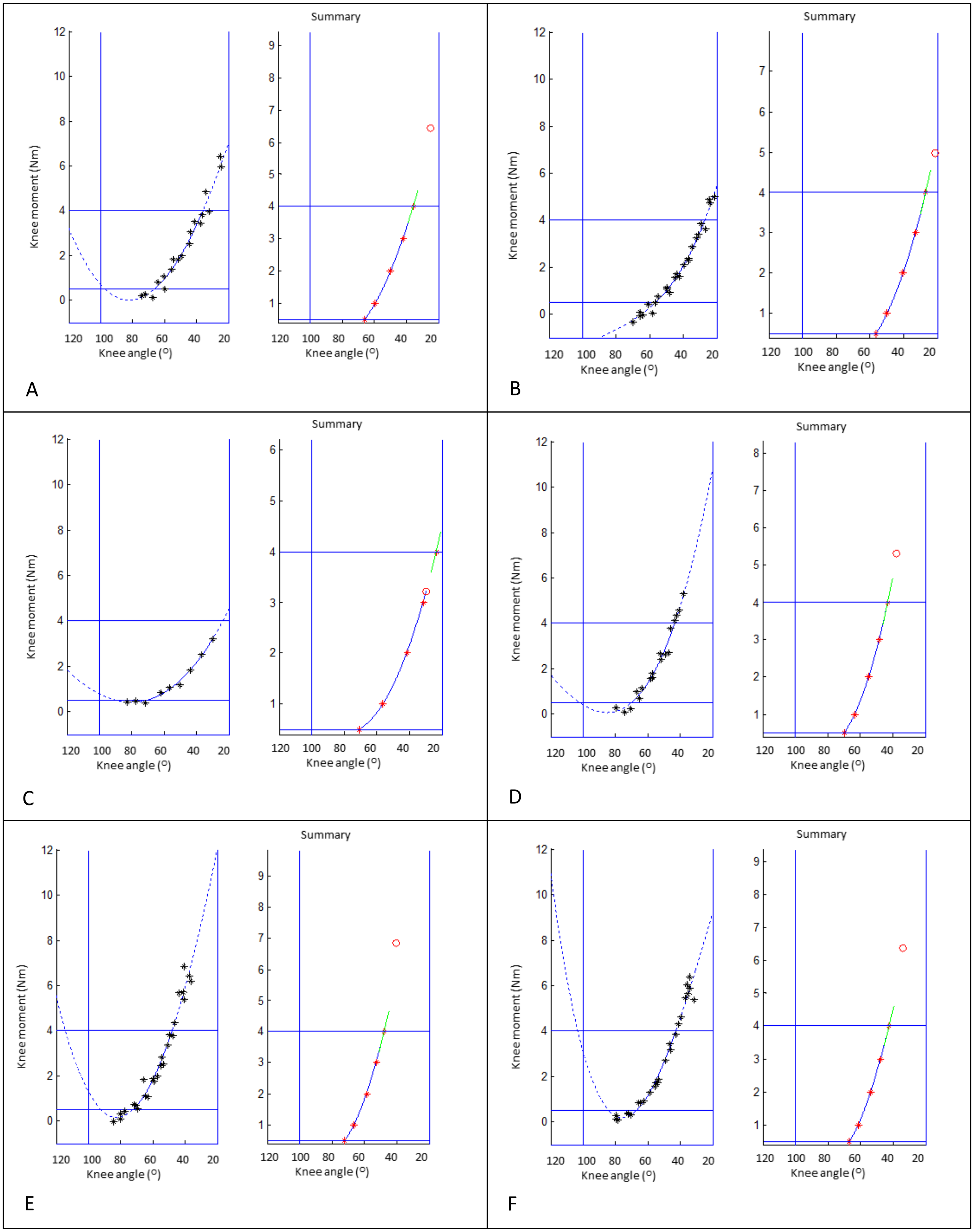
Summary
Knee moment (Nm)
Knee angle (°)
A
B
C
D
E
F

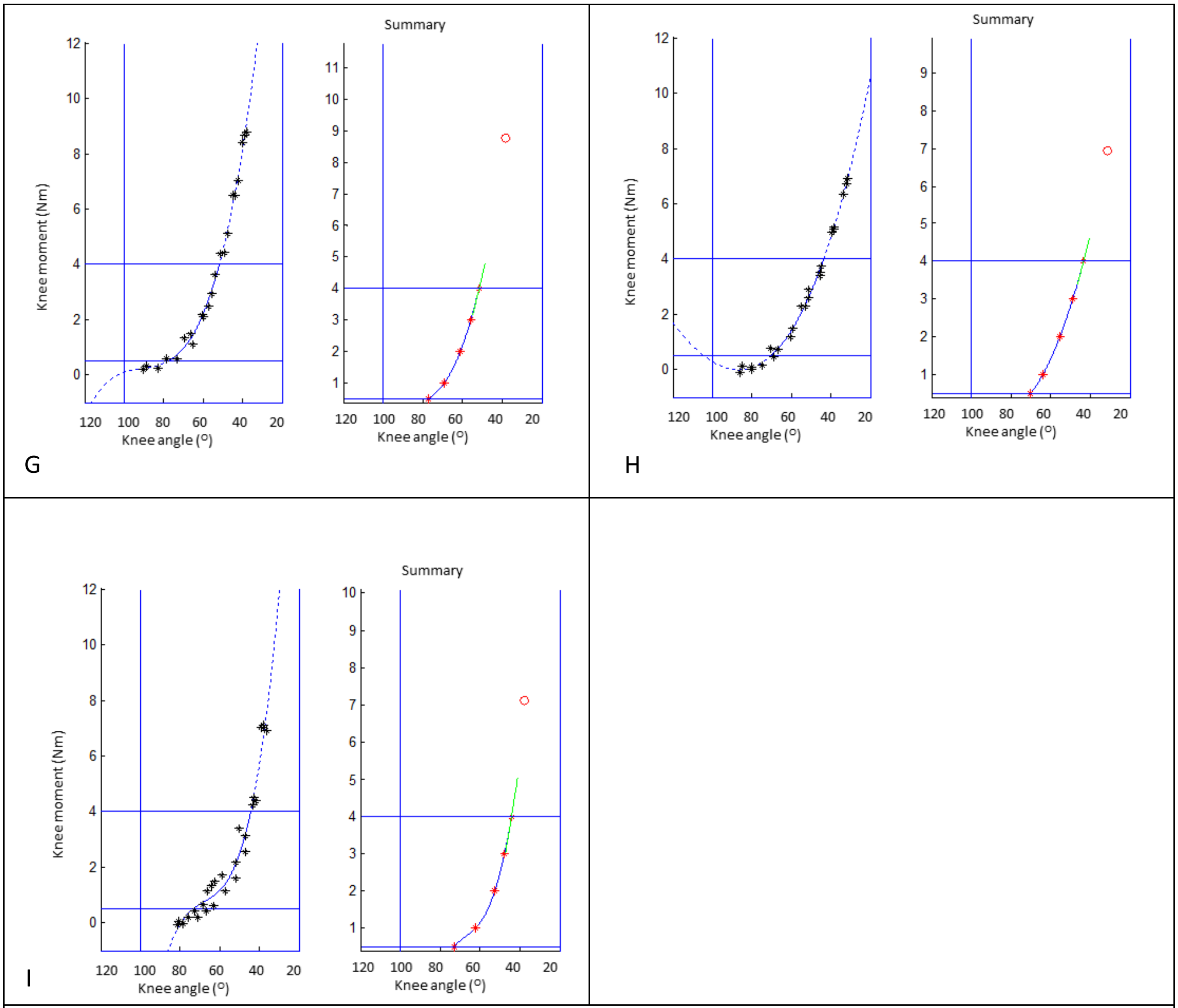

Fig. 7. **Individual data of nine typically developing children– pooled data of three repetitions and summary plot (A-I):** Black *=individual measured data points after exclusion of data points beyond EMG-threshold. Blue dashed line=fitted function (i.e. 3rd order polynomial function). Blue solid line=fitted line within the range used for comparison (i.e. knee angles corresponding to 0.5-4 Nm). Red *= derived estimates of the fit for statistical comparison at 0.5, 1, 2, 3 and 4 Nm. Red ^{o}= maximum measured angle. Green line = tangent line at 4 Nm (slope at 4 Nm is derived for comparison).
